# A Global Assessment of Plant and Animal Community Responses to Forest Management Over Time

**DOI:** 10.1111/gcb.70279

**Published:** 2025-06-16

**Authors:** Hanneke van 't Veen, Koen Kuipers, Aafke Schipper, Alexandra Marques, Mart‐Jan Schelhaas, Rob Alkemade

**Affiliations:** ^1^ Netherlands Environmental Assessment Agency Global Sustainability Den Haag the Netherlands; ^2^ Radboud University, Radboud Institute for Biological and Environmental Sciences Department of Environmental Science Nijmegen the Netherlands; ^3^ Sustainable Forest Ecosystems Wageningen University Wageningen the Netherlands; ^4^ Earth System and Global Change Group Wageningen University Wageningen the Netherlands

**Keywords:** abundance, forestry, intactness, mean species abundance (MSA), restoration, richness, threatened species

## Abstract

Transitions to forest management that mitigate negative effects of forest use on biodiversity are required to bend the curve of biodiversity loss. To facilitate such transitions, it is vital to understand the effects of different forest management practices on biodiversity. In this study, we analyzed observation data of 182 studies (312,453 abundance values) from three global biodiversity databases to estimate the effects of seven types of forest management on plant and animal (i.e., insects and vertebrates) biodiversity over time, and on threatened animals. We captured biodiversity in four distinct metrics (i.e., quantifiable measures of community composition): (i) intactness, (ii) relative species richness, (iii) compositional similarity, and (iv) relative total abundance, all calculated as the difference in biodiversity between managed and undisturbed forests. Overall, we find larger effects of forest management on intactness and similarity compared to richness and abundance. This suggests that forest management leads to a loss of species that specifically occur in undisturbed forests but that this decline is partially offset by an influx of species from other areas. We find that extensive forest management, such as selective cutting and agroforestry, supports higher levels of overall and threatened biodiversity than intensive management, such as forest and perennial tree crop plantations. We also find significant increases in animal community intactness and similarity in agroforests and forest plantations over 40 and 80 years since establishment, respectively, but do not find this for relative species richness and total abundance. This indicates that animal communities in these management systems become increasingly similar to those of undisturbed forests. Overall, our results highlight the potential of selective cutting and agroforests to mitigate biodiversity loss compared to more intensive systems, as well as the potential of longer rotation periods in forest plantations to increase habitat availability for species specifically adapted to undisturbed forests.

## Graphical abstract

Written Summary: We assessed the effects of seven forest management systems on biodiversity over time. We find that biodiversity responds more negatively to intensive than extensive forest management, but we also find that biodiversity can recover over time, particularly in intensively managed plantations and agroforests.

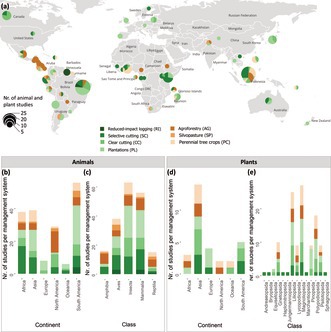

## 1 Introduction

Global extraction of roundwood and wood fuel, as well as the conversion of forests into agriculture, results in deforestation (FAO 2020a) and extensive forest degradation (Matricardi et al. 2020), with grave consequences for biodiversity (IPBES 2019). To bend the curve of biodiversity loss, it is insufficient to protect biodiversity in conservation areas alone (Leclère et al. 2020). There is also a need for more sustainable production and consumption of natural resources outside of protected areas (Laurance et al. 2012). That is why the Convention of Biological Diversity set target 1 of the Kunming‐Montreal Global Biodiversity Framework to facilitate management transitions that mitigate biodiversity loss on all working land by 2030 (Convention on Biological Diversity (CBD) 2022). In response, multiple countries and regions initiated transitions to forest management that mitigate the effects of forest use on biodiversity (MacDicken et al. 2015). For instance, the new EU forest strategy for 2030 of the European Union (EU) promotes a transition to more sustainable forest management that benefits biodiversity (European Commission 2021). Yet, to effectively enable such transitions, there is a need to identify management that can help mitigate forest use‐related biodiversity loss.

Previous global studies on the effects of forest management on biodiversity revealed that some forest management systems (i.e., sets of actions to achieve objectives of wood or food production from forests) are less harmful to biodiversity than others (Chaudhary et al. 2016). In forests managed for wood production, biodiversity is often higher in extensively managed areas, where natural forests are selectively cut and allowed to regenerate naturally, than in intensively managed areas, where forests are clear cut and planted (Chaudhary et al. 2016; Crouzeilles et al. 2017), in particular when non‐native species are planted (Vu Ho et al. 2023). In forests managed for food production, extensive production in agroforests (e.g., cocoa agroforests) usually has smaller effects on biodiversity than intensive production in perennial tree crop plantations (e.g., oil palm plantations) (Bohada‐Murillo et al. 2020; Deheuvels et al. 2012; Yahya et al. 2022). Smaller impacts of extensive than intensive forest management on biodiversity are related to the generally higher heterogeneity of vegetation structure (i.e., the three‐dimensional distribution of plants in the forest ecosystem) and composition under extensive than intensive management (Atlegrim and Sjöberg 2004). As a result, more food and shelter is available for species under extensive than intensive forest management (Atlegrim and Sjöberg 2004).

Even though extensive forest management generally has smaller effects on biodiversity than intensive management, the effects tend to vary between different aspects of biodiversity (e.g., species richness, abundance, and community composition) (Chaudhary et al. 2016; Lelli et al. 2019; Paillet et al. 2010). This is related to the varying effects of forest management on food and shelter resources that are preferred by different species groups (Paillet et al. 2010). For instance, species richness is often affected less than species composition in both clear‐cut and selectively cut forests (Crouzeilles et al. 2016; Dunn 2004; Xu et al. 2015), birds often respond more negatively to clear cutting than mammals (Chaudhary et al. 2016), and threatened species are often more vulnerable to human disturbances than non‐threatened species (Monks and Burrows 2014) and disproportionately affected by natural forest loss (Betts et al. 2017).

The effects of forest management on biodiversity can also be time‐dependent (Tudge et al. 2023). Some studies show that small management‐related disturbances can continue to affect biodiversity for decades (Gatti et al. 2015; Langridge et al. 2023), while other studies show that biodiversity in managed forests can recover rapidly upon forest regrowth (Tudge et al. 2023). For instance, while the effect of selective cutting on biodiversity is often relatively small compared to that of plantations, the recovery of biodiversity is slow under selective cutting due to long‐lasting effects on vegetation structure and composition (Gatti et al. 2015; Langridge et al. 2023). In contrast, despite the large initial impact of clear cutting and tree planting, biodiversity can recover rapidly over time in forest plantations (Hua et al. 2022; Tudge et al. 2023). This may be related to increases in vegetation structural complexity and tree diversity over time upon forest growth in plantations (Tudge et al. 2023).

At present, three important knowledge gaps remain to identify forest management systems that can help mitigate biodiversity loss related to forest use. First, no global study has yet compared the time‐dependent responses of biodiversity across different forest management systems. For instance, changes in biodiversity over time have not yet been compared between extensive and intensive forest management, nor have they been compared between forest management for food and wood production. Time‐dependent responses of biodiversity are relevant to consider when assessing the potential of forest management to mitigate biodiversity loss, as disregarding these responses may result in over‐ or underestimations of the effect of forest management on biodiversity. For instance, disregarding the potential of biodiversity recovery in forest plantations could result in an overestimation of biodiversity loss. Second, global studies often focus on only a single biodiversity metric (usually species richness) (Chaudhary et al. 2016; Lelli et al. 2019; Paillet et al. 2010), while different aspects of biodiversity may respond in varying ways to forest management. For example, if only the effects of forest management on species richness are assessed, changes in the types of species and their abundance over time remain unnoticed. Third, and finally, no global study has yet shown the effects of forest management on threatened species composition. Hence, it remains unclear if transitions in forest management could mitigate threatened species loss in response to forest use.

In this study, we aimed to determine the effects of different forest management systems on alpha biodiversity (i.e., insects, vertebrates, and plants) across the globe. We aimed to capture biodiversity responses to forest management in different metrics, namely biodiversity intactness, relative species richness, compositional similarity, and relative total abundance. To do this, we combined three existing datasets on biodiversity responses to land use and expanded them with information on forest management. We then estimated the overall and time‐dependent effects of forest management systems on biodiversity using linear mixed‐effect models. The results of this study can be used to identify forest management systems that are least harmful to biodiversity, while considering different metrics and taxonomic groups, as well as the potential for biodiversity recovery over time. Further, the results of this study may be used to parameterize global biodiversity models to run scenarios that may reveal how forest management systems may be best introduced and combined over space and time to mitigate biodiversity loss (Kok et al. 2018, 2023).

## 2 Methodology

### 2.1 Databases

We obtained data from three existing global biodiversity databases (i.e., PREDICTS (Hudson et al. 2014, 2017; Lawrence et al. 2016), GLOBIO (Alkemade et al. 2009), and a database compiled by Kuipers et al. (2023)) that contain data on animal (i.e., insects, amphibians, reptiles, birds, and mammals) and plant species populations or assemblages in sites subject to different land use and management types. We chose these three databases because they are, to our knowledge, the only global databases that contain paired observations of communities in disturbed sites or sites subject to land use as compared to reference sites (e.g., undisturbed natural vegetation) (Alkemade et al. 2009; Hudson et al. 2014; Kuipers et al. 2023). All databases were compiled through literature review and subsequent data extraction (Alkemade et al. 2009; Hudson et al. 2014; Kuipers et al. 2023).

Each of the three databases includes taxonomic, methodological (e.g., describing the data collection approach), and land use data. Additionally, each database includes species‐ to genus‐level abundance data from individual empirical scientific field studies across the globe. Each primary study (i.e., paper or report) in the databases is a single source. Each source may contain one or more studies, distinguished from each other based on their location and/or the year in which data were gathered. Within each study, data were collected from at least two sites within a given study area (e.g., in blocks or across transect lines), including at least one site subject to land use and one reference site. We defined reference sites as forests with no clearly visible indications of human activities for at least 80 years. Our definition of reference sites is in line with the FAO definition of primary forests, that is, “naturally regenerating forest of native tree species, where there are no clearly visible indications of human activities, and the ecological processes are not significantly disturbed” (FAO 2020b). We used a threshold of 80 years because it corresponds to the threshold conventionally used to distinguish young from mature forests (Keith et al. 2009; Liu et al. 2014; Odum 1969). Combined, the databases include 860 studies with per‐species abundance data of which 602 were from PREDICTS, 146 were from GLOBIO, and 171 were from Kuipers et al. (2023).

#### 2.1.1 Database Expansion and Forest Management Classification

We expanded the three databases with data on forest management for wood and food production. More specifically, we returned to the original sources to complement the databases by adding data for different aspects of management, including regeneration and tree harvesting strategies. We distinguished seven forest management systems: reduced‐impact logging, selective cutting, clear cutting and unassisted regrowth, forest plantations, agroforests, silvopasture plantations, and perennial tree crop plantations (see Table 1 for structured descriptions of all systems). These systems included the most common forest management systems found globally (Chaudhary et al. 2016; Crouzeilles et al. 2016). We used the conceptual framework of Trentanovi et al. (2023), which was developed to consistently study the effects of forest management on biodiversity, to define our management systems (Table 1). We adopted this framework because it standardizes forestry and forest structure terms and definitions to allow for an improved understanding of research outputs and a better comparison across biodiversity studies (Trentanovi et al. 2023). We distinguished between systems predominantly focusing on wood production and systems predominantly focusing on food production. In case both wood and food production may occur in a given forest management system (e.g., in silvopastures), we categorized the system under food production systems. Our definition of agroforests applies to the types of agroforestry that occur in the tropics (e.g., coffee plantations or subsistence agroforests). Hence, our biodiversity estimates for this system did not reflect the effect of agricultural systems that are often considered agroforests in temperate regions, such as orchards and trees planted in rows between crops.

**TABLE 1:** Names and descriptions of forest management systems distinguished in this study based on Trentanovi et al. (2023).

System focus	Predominantly focused on wood production				Predominantly focused on food production		
Management system	**Reduced‐impact logging (RI)**	**Selective cutting (SC)**	**Clear cutting and regrowth (CC)**	**Forest plantations (PL)**	**Agroforests (AG)**	**Silvopasture (SP)**	**Perennial tree crops (PC)**
	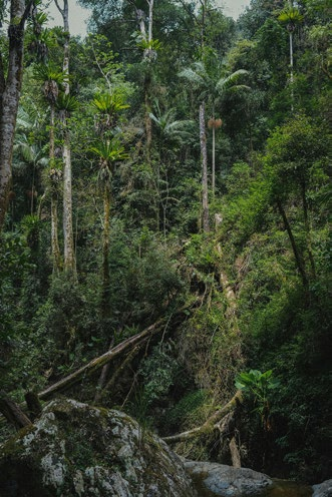	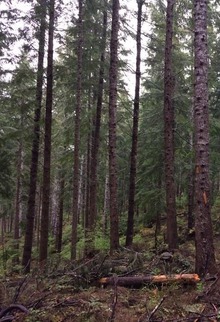	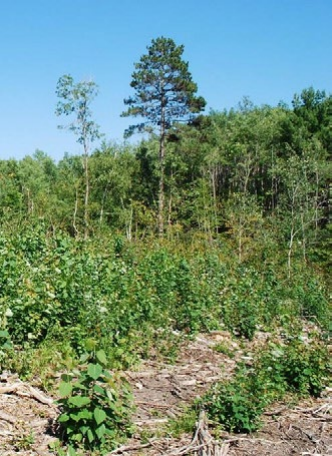	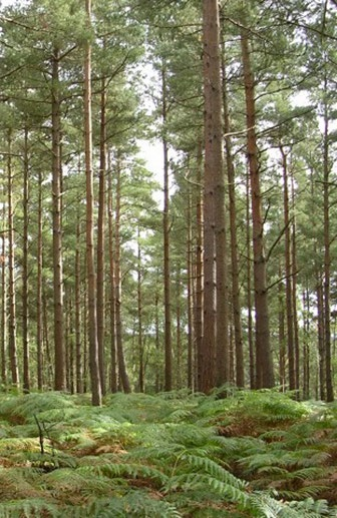	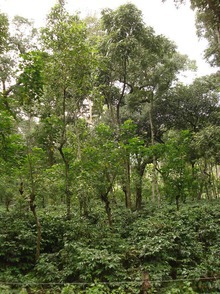	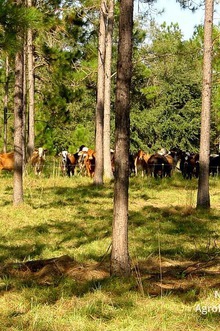	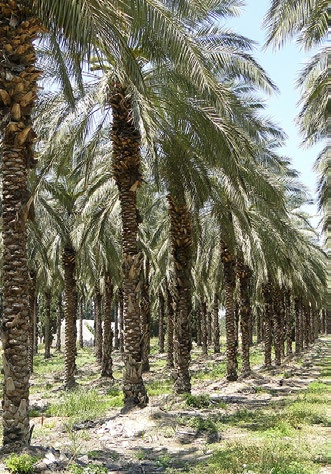
Management activities	Harvesting of a small number of single trees according to strict harvesting guidelines that are intended to minimize effects of logging on biodiversity	Harvesting of single trees or patches of trees across the forest, selected according to (predefined) harvesting rules and/or preferences	Harvesting of an entire stand of trees within a forest area in a single operation	Land on which one or multiple tree species, either native or exotic, are planted or seeded, which are harvested in a single operation	Crop production underneath tree canopy. Trees may be harvested and planted or seeded on occasion	Livestock production underneath tree canopy. Trees may be harvested and planted or seeded on occasion	Tree planting plantations for crop production
Regeneration type	Natural regeneration	Natural regeneration	Natural regeneration	Planting or direct seeding	Natural regeneration, planting or direct seeding	Natural regeneration, planting, or direct seeding	Planting or direct seeding
Time since last intervention	Time since the last tree harvesting event	Time since the last tree harvesting event	Time since the last tree harvesting event	Time since the last tree harvesting and planting event	Time since establishment (i.e., time since crop or tree planting)	Time since establishment (i.e., livestock introduction or tree planting)	Time since establishment (i.e., perennial tree crop planting)
Vegetation structural complexity^a^	Heterogeneous	Heterogeneous	Homogeneous	Homogeneous	Heterogeneous	Heterogeneous	Homogeneous
Stand vertical Structure^b^	Full‐storied or multi‐layered	Full‐storied or multi‐layered	Single‐storied	Single‐storied	Two‐storied or multi‐layered	Single‐storied or multi‐layered	Single‐storied
Tree species richness	Multiple tree species	Multiple tree species	Multiple tree species	Mostly single tree species	Multiple tree species	Multiple tree species	Mostly single tree species
Tree species nativeness	Native, unless invaded by non‐native species	Native, unless invaded by non‐native species	Native, unless invaded by non‐native species	Planted species are either non‐native or native	Native, if established in natural forests, unless invaded by non‐native species. Non‐native or native trees are planted if established in non‐forested land	Native, if established in natural forests, unless invaded by non‐native species. Non‐native or native trees are planted if established in non‐forested land	Planted species are either non‐native or native
Canopy gaps	Scattered gaps, smaller in size as those under SC	Scattered gaps, bigger in size as those under RI	No gaps	No gaps	Scattered gaps	Scattered gaps	No gaps
Active management^c^	No; natural forest regrowth without human intervention	No; natural forest regrowth without human intervention	No; natural forest regrowth without human intervention	Yes; stand management (e.g., thinning)	Yes; continuous crop production and crop harvesting	Yes; continuous livestock tending	Yes; continuous crop production and crop harvesting

*Note:* The following definitions of forest structure‐related terms were used. The definitions of forestry and forest structure‐related terms can be found in this Table and Table 2 of Trentanovi et al. (2023).

^a^ *Vegetation structural complexity*: The three‐dimensional distribution of plants in the forest ecosystem (Coverdale and Davies 2023). *Heterogeneous structure*: High variability in the three‐dimensional distribution of plants in the ecosystem. *Homogeneous structure*: Low variability in the three‐dimensional distribution of plants in the ecosystem.

^b^ *Stand vertical structure*: The height stratification of tree layers in the forest (Trentanovi et al. 2023). Stand vertical structure is an aspect of vegetation structural complexity. *Full‐storied*: A mixture of trees of almost every possible height (Lundqvist 2017). *Two‐storied*: The height distribution of trees is bimodal (Lundqvist 2017). *Multi‐layered forests*: The height distribution of trees falls between two‐storied and full‐storied (Lundqvist 2017). *Single‐storied*: All trees have a similar height (Lundqvist 2017).

^c^ Active management indicates whether human management interventions occur following tree harvesting or tree planting, as opposed to passive management where forests regenerate by themselves with no or very limited human interference until they are ready to be harvested.

From the original sources, we also obtained information on the time since harvesting of trees (in years) for reduced‐impact logging, selective cutting, and clear‐cutting systems (previous rounds of cutting could have occurred), and time since the establishment (in years) of forest plantation, agroforest, silvopasture, and perennial tree crop plantation systems. In the case of forest plantations, time since establishment indicated the time that passed since trees were planted (prior rounds of planting could have occurred). Data on time since harvesting or establishment were available for approximately half of the studies on animal biodiversity.

We completed the species‐level entries in the database with the threat status of species. For this purpose, we downloaded the IUCN Red List for plants, insects, amphibians, reptiles, birds, and mammals (https://www.iucnredlist.org/, last accessed 19 July 2024). The IUCN Red List includes, among others, the binominal scientific names of threatened species and their risk of global extinction, ranging from least concern to extinct (IUCN 2024). We merged the IUCN Red List with our combined biodiversity database by matching the binominal names of the species in our database with those of threatened species in the IUCN Red List.

#### 2.1.2 Data Availability and Distribution

Of the 860 studies in the three databases, 182 studies, derived from 123 publications, included data gathered in sites under one of the forest management systems we distinguished in this study and corresponding reference sites. From these 182 studies, 157 analyzed animal diversity and 25 analyzed plant diversity.

**FIGURE 1:**
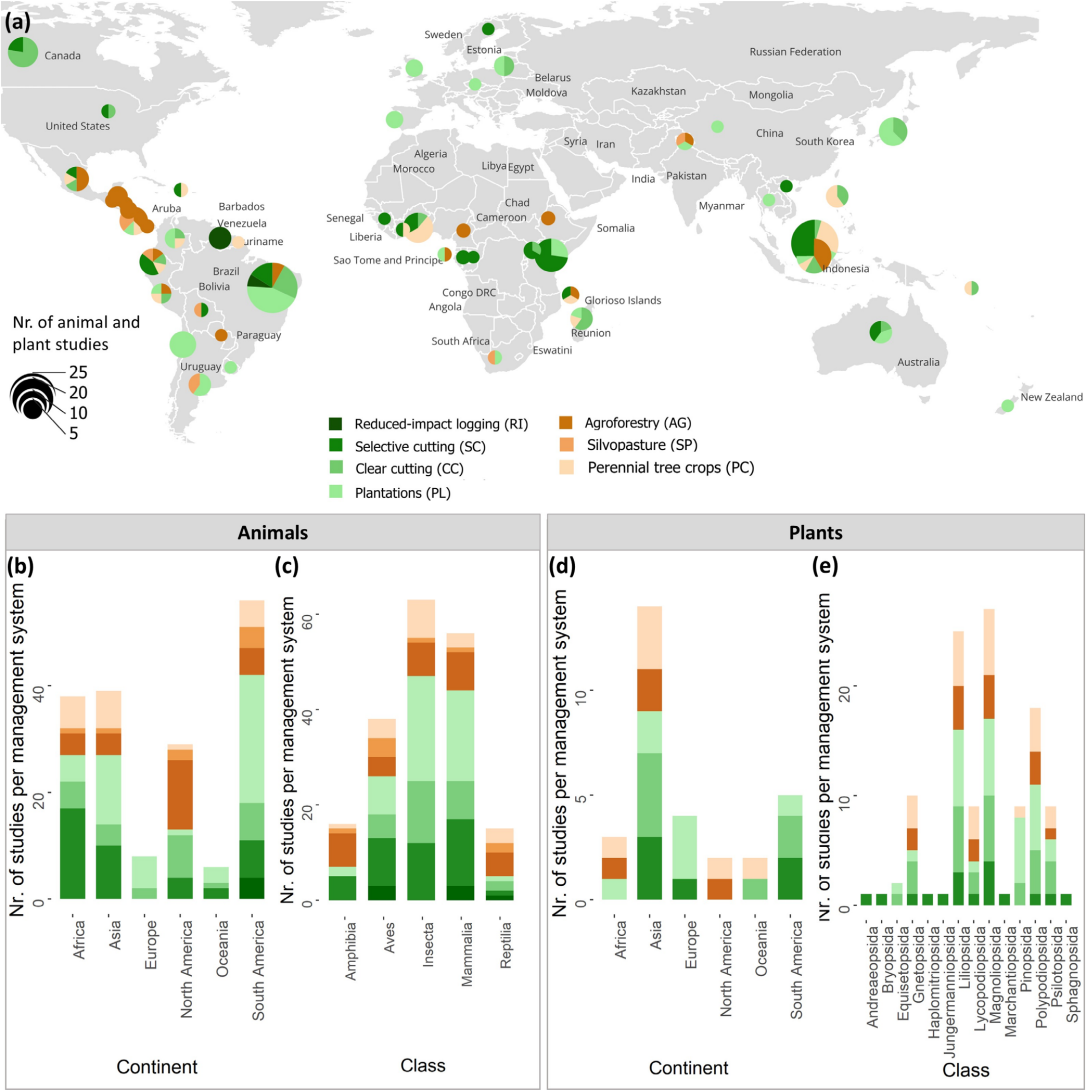
Spatial distribution and numbers of included studies. (a) The global distribution of empirical biodiversity studies categorized by forest management system. The wood production systems are colored in greens and the food production systems in browns. The sizes of the pie charts indicate the number of studies per country included in the databases. (b) Animal biodiversity studies per continent categorized by forest management system. (c) Animal biodiversity studies per taxonomic class categorized by forest management system. (d) Plant biodiversity studies per continent categorized by forest management system. (e) Plant biodiversity studies per taxonomic class categorized by forest management system. Each study can contain multiple management systems and cover multiple taxonomic classes. Map lines delineate study areas and do not necessarily depict accepted national boundaries.

In total, the database contained 159,148 abundance values of 9240 animal species or genera and 153,305 abundance values of 4917 plant species or genera obtained in sites subject to one of the management systems and corresponding reference sites. Most studies originated from South America (*N*_animals_ = 53, *N*_plants_ = 6), followed by Asia (*N*_animals_ = 33, *N*_plants_ = 9), Africa (*N*_animals_ = 34, *N*_plants_ = 3), North America (*N*_animals_ = 25, *N*_plants_ = 1) (primarily Central America), Europe (*N*_animals_ = 6, *N*_plants_ = 5), and Oceania (*N*_animals_ = 6, *N*_plants_ = 1) (Figure 1a–e). Studies for all seven management systems were available for South America. For North America, Asia, and Africa, studies of six management systems were included, and for Oceania and Europe, only studies of three and two systems were available, respectively (Figure 1b,d). In South America, Asia, Oceania, and Europe, plantation systems were best represented in the databases, followed by selective cutting systems in South America and Asia. In North America, agroforests were best represented, while in Africa, most studies focused on selective cutting.

Data availability differed among animal taxonomic classes. The databases included the highest number of studies for insects (63), followed by mammals (56), birds (38), amphibians (16), and reptiles (15) (Figure 1). For animals, data were available on at least five management systems for all taxonomic classes. Less data were available for plants (30 studies); the databases only contained plant data gathered in five management systems: agroforests, clear cutting and regrowth, perennial tree crop plantations, forest plantations, and selective cutting. The most abundant plant classes were Liliopsida, Magnoliopsida, and Polypodiopsida.

### 2.2 Biodiversity Metrics and Correlation Analysis

We measured the effects of forest management on alpha animal and plant biodiversity using four biodiversity metrics: intactness (IN), relative species richness (SR), compositional similarity (SI), and relative total abundance (TA). Prior to calculating these metrics, we averaged the abundances of each species or genus across the sites (e.g., plots or transects) of each study per forest management system and for corresponding reference sites, respectively. We note that while the abundance values in our databases include both species and genus‐level measurements, we refer to species from here on for the ease of reading.

We calculated biodiversity intactness per study and per forest management type as mean species abundance (MSA), a measure of local biodiversity intactness based on species‐specific differences in abundances between managed and reference sites (Alkemade et al. 2009; Kuipers et al. 2023; Schipper et al. 2020). MSA reflects how similar the species composition of managed sites is to that of reference sites. We first divided the abundances of species found under a certain management system by those of corresponding reference sites, providing us with abundance ratios (i.e., response ratios) per management system per species. If a species occurred in the managed sites but not in the reference sites, we removed this species. If the abundance of a species in managed sites was higher than that of corresponding reference sites, the value was set at 1 (maximum intactness). Finally, we averaged the resulting ratios across each study and divided the average by the number of species found in reference sites. Thus, we calculated intactness (IN) for each study and management system as follows:

(1) $${\text{IN}}_{m,i}=\frac{\sum_{s}^{N_{r}}\min\left(A_{i,s,m}/A_{i,s,r},1\right)}{N_{r}}$$

where, *A* represents the abundance of species (*s*) in sites subject to a certain management system (*m*) or in corresponding reference sites (*r*) for study (*i*), and *N* is the number of species in the reference sites. Truncating relative species abundance at 1 (in the numerator of Equation 1) ensured that possible increases in abundance in response to management did not mask abundance losses of other species (Alkemade et al. 2009; Schipper et al. 2020).

We assessed compositional similarity between managed and reference sites using the Sørensen Similarity Index (Sørensen 1948). Per study and management system, we determined the number of species that occurred in both managed and corresponding reference sites, as well as the number of species that differed, respectively. Hereafter, we divided the number of overlapping species times two by the total number of species that overlapped and differed times two. Thus, we calculated the Sørensen Similarity Index (SI) for each study and management system as follows:

(2) $${\mathrm{SI}}_{m,i}=\frac{2*T_{m,r,i}}{\left(2*T_{m,r,i}+F_{m,r,i}\right)}$$

where, *T* represents the number of species common to sites subject to a certain management system (*m*) and corresponding reference sites (*r*), and *F* is the count of species that differ between sites subject to a certain management system and corresponding reference sites in study (*i*).

We evaluated the effects of forest management on relative species richness by computing the log‐response ratio between the species richness observed in management sites and that of reference sites. To derive relative species richness, we determined the total number of species found in managed sites for each study, as well as the total number of species found in corresponding reference sites, respectively. We then divided the number of species found for specific forest management sites per study by that of corresponding reference sites and took the natural log to adjust for the right‐skewed distribution of the response ratios (Hedges et al. 1999). Thus, we calculated relative species richness (SR) for each management system in each study as follows:

(3) $${\mathrm{SR}}_{m,i}=\ln\left(\frac{S_{m,i}}{S_{r,i}}\right)$$

where, *S* denotes the number of species in sites subject to a certain management system (*m*) or in the corresponding reference sites (*r*) for each study (*i*).

Finally, we evaluated the effect of forest management on relative total abundance. Per study, we first summed the abundances of all species in sites subject to a certain management system and the abundances of all species in the corresponding reference sites. Then, we divided the summed abundance of managed sites by that of the corresponding reference sites per study and took the natural log to adjust for the right‐skewed distribution of the response ratios (Hedges et al. 1999). Thus, we calculated relative total abundance (TA) for each management system per study as follows:

(4) $${\mathrm{TA}}_{m,i}=\ln\left(\frac{\sum_{s}^{M}A_{i,s,t}}{\sum_{s}^{N}A_{i,s,p}}\right)$$

where, *A* is the abundance of species (*s*) in sites subjected to a certain management system (*m*) or the corresponding reference sites (*r*) in each study (*i*), and *M* and *N* represent the total number of species in the managed and reference sites, respectively.

We chose the four metrics because previous studies show that a combination of species richness, abundance and intactness‐related biodiversity metrics is needed to capture biodiversity changes in response to anthropogenic pressures (Kuipers et al. 2023; Santini et al. 2017; Vačkář et al. 2012). Intactness indicates how closely managed sites compare to those of reference sites in terms of the abundance of species present in reference sites, while similarity reflects the overlap in species composition between managed sites and reference sites. Therefore, a comparison between intactness and similarity reveals whether a loss of intactness is mainly due to a loss of species specifically adapted to undisturbed forests or rather a reduced abundance of those species. Comparing intactness and similarity with relative species richness provides an indication of whether a transition from undisturbed to managed forests results in a shift from species specifically adapted to undisturbed forests to species that occur in other areas, or whether an overall loss of species occurs. Finally, a comparison between intactness and relative total abundance reveals whether the abundances of species specifically adapted to undisturbed forests decline or whether forest management causes a significant loss in species abundances overall.

After having calculated the metric values per management system and study, we conducted principal component analyses (PCA) and produced Spearman correlation matrices to assess correlations between biodiversity intactness, compositional similarity, relative species richness, and relative total abundance for animals and plants overall, respectively, as well as for insect, bird, herpetofauna, and mammals separately. The outcome of these analyses provided an indication of the complementarity and redundancy of our biodiversity metrics.

### 2.3 Hypothesized Effects of Forest Management on Biodiversity

We hypothesized that biodiversity mainly responds to management‐induced changes in vegetation composition and structural complexity (i.e., three‐dimensional distribution of plants in the forest ecosystem), because we expected these changes to affect food and shelter provisioning for species (Coverdale and Davies 2023) (Table 2). Prior studies show positive relationships between biodiversity and indices of vegetation structural complexity, such as the vertical diversity index and relative height (Coverdale and Davies 2023; Heidrich et al. 2023; Tews et al. 2004). Additionally, high tree diversity can positively influence biodiversity (Ampoorter et al. 2020), while the planting of non‐native tree species can negatively affect biodiversity (Vu Ho et al. 2023). Therefore, we hypothesized smaller effects of forest management on biodiversity when the vegetation structure and composition of managed forests are heterogeneous and more closely resemble that of undisturbed forests. Accordingly, we expected that biodiversity is less affected by selective cutting and clear‐cutting followed by natural regrowth of native tree species than by the planting of non‐native trees to create timber or perennial tree crop plantations. Hereby, it is important to note that there are gradients in vegetation structural heterogeneity and composition within managed and undisturbed forests. For instance, undisturbed forests can range from homogeneous stands composed of only one or two tree species, such as beech dominated primary forests (Kozák et al. 2018), to highly heterogeneous stands with high numbers of tree species and a naturally full‐storied canopy, such as tropical rain forests (Lowman and Moffett 1993). Selective cutting may have smaller effects on the vegetation structural heterogeneity and tree composition in more homogeneous undisturbed forests than in undisturbed forests with high numbers of tree species and a naturally full‐storied canopy.

**TABLE 2:** Summarized hypothesized effects of forest management on animal and plant biodiversity and general findings. A more detailed overview of the hypotheses and findings can be found in Table S8.

	Management	Hypothesized effects on biodiversity	General findings
Predominantly wood production	Reduced‐impact logging	Biodiversity is only slightly lower under reduced‐impact logging than reference sites because of the, by design, small effect of reduced‐impact logging on vegetation structure and composition (Bicknell et al. 2015; Schwartz et al. 2012) Selective harvesting of trees under reduced‐impact logging only slightly affects tree diversity and composition (Schwartz et al. 2012), yet it negatively influences intactness and similarity across taxonomic groups	Slight, yet significantly, lower animal intactness and similarity under reduced‐impact logging than in reference sites across taxonomic groups Similar richness and total abundance under reduced‐impact logging as reference sites across taxonomic groups
	Selective cutting	Relative to reference sites, selective cutting generally has larger effects on vegetation structure and composition than reduced‐impact logging (Imai et al. 2012), as generally more trees are cut under selective logging and less attention is paid to minimizing effects of logging on biodiversity (Pereira et al. 2002) Selective harvesting of trees significantly affects tree diversity and composition (Imai et al. 2012), as well as the vertical structure of the canopy (Okunda et al. 2003), which negatively influences biodiversity across metrics and taxonomic groups	Slight, yet significantly, lower intactness and similarity under selective logging than reference sites across taxonomic groups Similar richness and total abundance under selective cutting as reference sites across taxonomic groups A slight increase in intactness over a period of 50 years after logging
	Clear cutting and regrowth	Clear cutting and regrowth systems have significantly lower biodiversity than reference sites across taxonomic groups and across metrics because of their homogeneous vegetation structure, including the single‐storied canopy (Chaudhary et al. 2016; Crouzeilles et al. 2016) Clear cutting and regrowth affects biodiversity significantly more negative than selective cutting because of the homogeneous vegetation structure and single‐storied canopy of regrowing clear‐cut forests (Xu et al. 2015) Animal biodiversity recovers over time along successional gradients following natural forest regrowth after clear cutting (Xu et al. 2015)	Significantly lower intactness and similarity in clear cutting systems than reference sites across taxonomic groups Similar richness and total abundance in clear cutting systems as reference sites across taxonomic groups, except for plants Intactness and similarity decline over 70 years after harvesting
	Forest plantations	Forest plantations have a significantly lower biodiversity than reference sites across taxonomic groups and biodiversity metrics because of their homogeneous vegetation structure, including their single‐storied canopy and generally low numbers of tree species (Chaudhary et al. 2016; Crouzeilles et al. 2016) Effects of forest plantations on biodiversity are larger than that of clear cutting and regrowth, relative to reference sites, because of their lower tree diversity and the higher chance that non‐native tree species occur (Wang et al. 2022) Animal biodiversity increases slightly with plantation age across taxonomic groups (Tudge et al. 2023) as a result of enhanced tree heights, slight increases in the heterogeneity of vegetation structure and an expected influx of tree species and understory vegetation over time (Eycott et al. 2006)	Significantly lower overall animal and plant biodiversity across biodiversity metrics compared to reference sites Similar richness and total abundance to that of reference sites for birds and herpetofauna, as well as similar richness for mammals as in reference sites Strong significant increases in intactness and similarity over an 80‐year period since forest plantation establishment
Predominantly food production	Agroforests	Biodiversity is lower in agroforests than reference sites because their vegetation structure, including canopy layering, is less heterogeneous than that of reference sites and their tree composition less diverse (Perry et al. 2016) Agroforests affect intactness and similarity more than richness and total abundance, as there is an influx of species that do not occur in reference sites in agroforests and increases in abundances of some species and declines in others (Harvey and González Villalobos 2007; Jose 2012) Animal biodiversity increases slightly over time since establishment as trees age (De Leijster et al. 2021)	Significantly lower intactness and similarity in agroforests than in reference sites across taxonomic groups Similar richness and total abundance in agroforests as in reference sites across taxonomic groups Significant increases in intactness and similarity over a 40‐year period since agroforest establishment
	Silvopasture	Biodiversity is lower in silvopastures than reference sites because their vegetation structure, including canopy layering, is less heterogeneous than that of reference sites and their tree composition less diverse (Orefice et al. 2017) Silvopasture affects intactness and similarity more than richness and total abundance, as there is an influx of species that do not occur in reference sites and increases in abundances of some species and declines in others (Orefice et al. 2017; Perez‐Alvarez et al. 2023) Relative to reference sites, silvopastures affect biodiversity slightly more than agroforests because of higher heterogeneity in canopy layers in agroforests as a result of crop production	Significantly lower intactness and similarity in silvopastures than reference sites across taxonomic groups Similar richness and total abundance in silvopastures as reference sites across taxonomic groups
	Perennial tree crops	Biodiversity is significantly lower in perennial tree crops than reference sites across taxonomic groups and biodiversity metrics because of their homogeneous vegetation structure, including their single‐storied canopy and generally low numbers of tree species (Savilaakso et al. 2014) Perennial tree crops have significantly larger effects on biodiversity than agroforests and silvopastures (Yahya et al. 2022), because of their more homogeneous vegetation structure, lower tree diversity, as well as the higher chance that non‐native tree species are planted (Wang et al. 2022) Biodiversity does not increase over time because vegetation structure and tree composition of perennial tree crops remain similar over time because of continuous management activities, such as weeding, thinning, and the application of pesticides and herbicides	Significantly lower intactness and similarity under perennial tree crops than reference sites across taxonomic groups Significantly lower intactness, similarity and richness in perennial tree crops than agroforests Limited and contrasting changes in biodiversity over 40 years since establishment

We also expected differences in the effect of forest management on biodiversity across metrics, species groups, and over time since harvesting or establishment (Table 2).

- *Biodiversity metrics*: We expected a smaller effect of management on species richness and total abundance than on community intactness and similarity, as influxes of species that do not inhabit reference sites can occur in response to forest management (Paillet et al. 2010). Additionally, forest management has been shown to increase the abundance of some species, while decreasing the abundance of others (Økland et al. 2003).
- *Species groups*: We hypothesized that we would find larger effects of forest management on threatened species than on overall biodiversity, because we expected threatened species to be more dependent on the vegetation structure and composition that is specific to undisturbed forests (Betts et al. 2017).
- *Changes over time*: We hypothesized to find limited changes in biodiversity over time in planted forests, such as timber and perennial tree crop plantations, because we expected the vegetation structure of these forests to remain homogeneous over time. Besides this, we hypothesized a slow biodiversity recovery in forests that regenerate naturally after wood harvesting because prior local studies showed that the effects of management on vegetation structure can persist for decades (Gatti et al. 2015). Additionally, we expected limited recovery of biodiversity over time under forest management that predominantly focuses on food production because of the frequent recurring disturbances related to the planting and harvesting of crops.

### 2.4 Effects of Forest Management on Biodiversity

#### 2.4.1 Model Types and Response Variables

We analyzed all data in R4.3.1 (R Core Team 2023). We visualized spatial data in QGIS (QGIS Development Team 2023). We estimated the effects of forest management on biodiversity and assessed how these effects change over time and across taxonomic groups and geographical regions through generalized linear mixed‐effect modelling, tailored to the conditional data distributions of the metrics. We built two types of generalized linear mixed‐effect models: (i) beta‐regression models with a logit‐link function for intactness and compositional similarity, using the Temperate Model Builder R‐function *glmmTMB* (Brooks et al. 2023), and (ii) Gaussian linear mixed‐effect models for relative species richness and total abundance, using the *lmer* function of the R‐package *lme4* (Bates et al. 2015). We used beta‐regression models to estimate intactness and compositional similarity because these metrics cannot be lower than 0 or higher than 1. Beta‐regression does not allow for values of 0 and 1 (intactness and compositional similarity can be 0 or 1); therefore, we transformed intactness and compositional similarity to values between 0 and 1 using the Smithson–Verkuilen transformation (Smithson and Verkuilen 2006). We used Gaussian linear mixed‐effect models to estimate relative species richness and total abundance because log‐response ratios are normally distributed (Hedges et al. 1999). We built models for all animal taxa together and models for all plant taxa together, as well as separate models for insects, birds, herpetofauna, and mammals. We grouped amphibians and reptiles together as herpetofauna because of their small sample sizes. Further, we built separate models for the relative species richness, compositional similarity and relative total abundance of threatened animals of the categories “vulnerable” to “critically endangered”.

We weighted the response variables by the square root of the total number of species per study that occurred in reference sites, reasoning that studies based on a larger sample of species produce more representative estimates of the effects of forest management on biodiversity than studies based on a smaller sample. This increased the influence of studies with abundance data on a large number of species on the biodiversity estimates and reduced the influence of those studies with abundance data on a limited number of species. We square root transformed the number of species to mitigate skewness in the data.

#### 2.4.2 Fixed Effects

We considered the following fixed effects in our models: (i) forest management system, (ii) time since tree harvesting or the establishment of the management system, (iii) continent, and (iv) taxonomic group.

##### 2.4.2.1 Forest Management

We built models with the forest management systems as fixed effects to estimate overall effects of each of the forest management systems on biodiversity. For each management system, the database includes biodiversity observations gathered at different forest development stages (e.g., 5, 10, or 20 years after wood harvest or establishment). Hence, the overall biodiversity estimates included the effects of temporal changes in biodiversity that may occur upon forest growth and/or continuous management.

##### 2.4.2.2 Time Since Harvesting or Establishment

We built models with time since tree harvesting or establishment as a fixed effect for each forest management system separately, in order to reveal potential management‐specific time dependencies in animal biodiversity responses. We only studied changes in animal biodiversity over time due to data limitations. We limited ourselves to management systems for which sufficient data were available (≥ 5 data points). We gathered sufficient data to assess biodiversity changes over time for selective cutting, clear cutting and regrowth, forest plantations, agroforests, and perennial tree crop plantations. For selective cutting and clear‐cutting and regrowth, the time variable reflected the number of years since tree harvesting (previous rounds of cutting could have occurred). For the remaining management systems, time reflected the number of years since establishment (see Section 2.1.1). We visually assessed the occurrence of non‐linear relationships between the biodiversity metrics and time since harvesting and establishment. In case we observed clear signs of non‐linearity, we assessed if a log transformation of time since harvesting or establishment significantly improved model performance based on the Bayesian Information Criterion (BIC). We considered the models with the lowest BIC values the best performing ones. Finally, we tested if adding time since harvesting or establishment as a fixed effect explains the variation in the biodiversity data significantly better than a model with only the intercept by comparing the BIC, as well as through ANOVA.

##### 2.4.2.3 Taxonomic Class and Continent

We built models with forest management systems and animal taxonomic classes together as additive fixed effects to test for potential differences in the responses of different taxonomic classes to forest management by comparing the BIC, as well as through ANOVA. We also built models with forest management systems and continents together as additive fixed effects to test for potential intercontinental differences in the response of biodiversity to forest management by comparing the BIC, as well as through ANOVA.

#### 2.4.3 Random Effects

For the overall animal diversity models, we added nested random effects of source‐study combinations (1|Source/Study) to account for potential differences among datasets and non‐independence of data. Hereby, the “Source” is the primary source (i.e., paper or report) from which data were included in the database, and “Study” refers to the respective study that was conducted by the researchers for each Source (see Section 2.1). For plant, insect, bird, herpetofauna, mammal, and threatened species diversity models, we only added the source (1|Source) as a random effect because for most sources only one study per source was conducted. We did the same for models that estimated changes in biodiversity over time since the establishment of perennial tree crops.

#### 2.4.4 Model Evaluation and Visualization

We conducted Omnibus tests (i.e., a likelihood‐ratio chi‐square test that estimates the significance of adding a fixed effect compared to a null model). We conducted Tukey post hoc tests with Bonferroni correction to estimate differences between forest management systems for wood production, as well as between management systems for food production for all animal taxa together and plants, using the function *glht* of the R package *multcomp* (Hothorn et al. 2024). We used post hoc tests to assess the difference between forest management systems in which wood was extracted from forests that were subsequently allowed to regenerate naturally (i.e., selective cutting, reduced‐impact logging, and clear cutting and regrowth) and systems in which wood was extracted from plantations (i.e., forest plantations). We repeated this to assess the difference between forest management systems in which food was produced under the canopy (i.e., agroforests and silvopasture) and those in which food was produced in plantations (i.e., perennial tree crop plantations). We did not compare management systems for wood production with those for food production because these systems do not fulfill the same function and, hence, are not interchangeable. To present our results, we back‐transformed the log‐response ratios of species richness and total abundance. We also back‐transformed intactness and similarity estimates based on logit‐link distributions.

## 3 Results

### 3.1 Correlations Between Biodiversity Metrics

The Spearman correlation assessment revealed that all biodiversity metrics were positively related to each other, with Spearman rank correlations (rho) between 0.1 and 0.7 for animals (all taxonomic groups combined) and between 0.2 and 0.9 for plants (Figures S1 and S2). The PCAs showed orthogonal orientations of the principal components for relative species richness and total abundance.

### 3.2 Effects of Forest Management on Biodiversity

#### 3.2.1 Intactness

**FIGURE 2:**
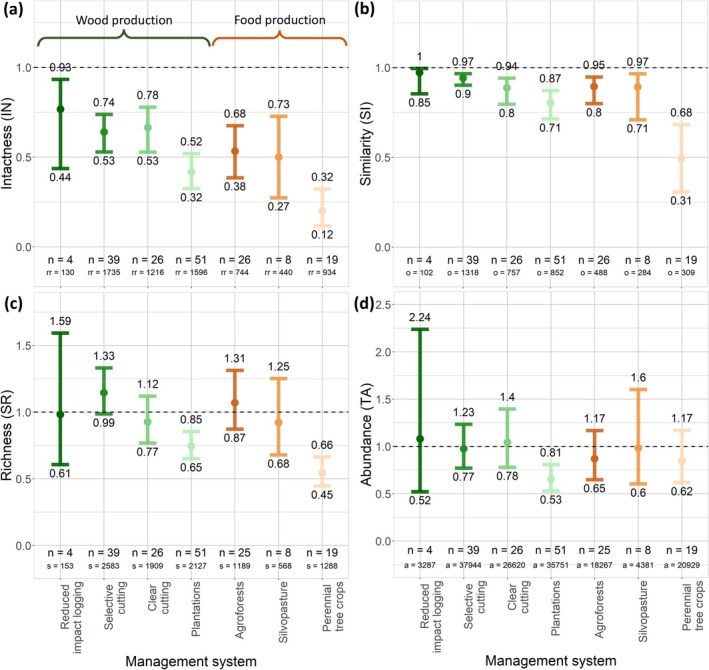
Effects of forest management on animal communities, expressed as four metrics: intactness (IN) (a), similarity (SI) (b), relative species richness (SR) (c), and relative total abundance (TA) (d). Each colored dot shows the back‐transformed mean estimate we derived from our mixed models, and the bars indicate the corresponding 95% confidence interval. The dotted line indicates the reference level (i.e., the biodiversity of the reference sites). *n* = number of studies per management type, rr = number of response values used to compute intactness, *o* = number of overlapping species between managed and reference sites, *s* = number of species occurring across the managed and reference sites, and *a* = number of abundance values used to compute relative total abundance.

**FIGURE 3:**
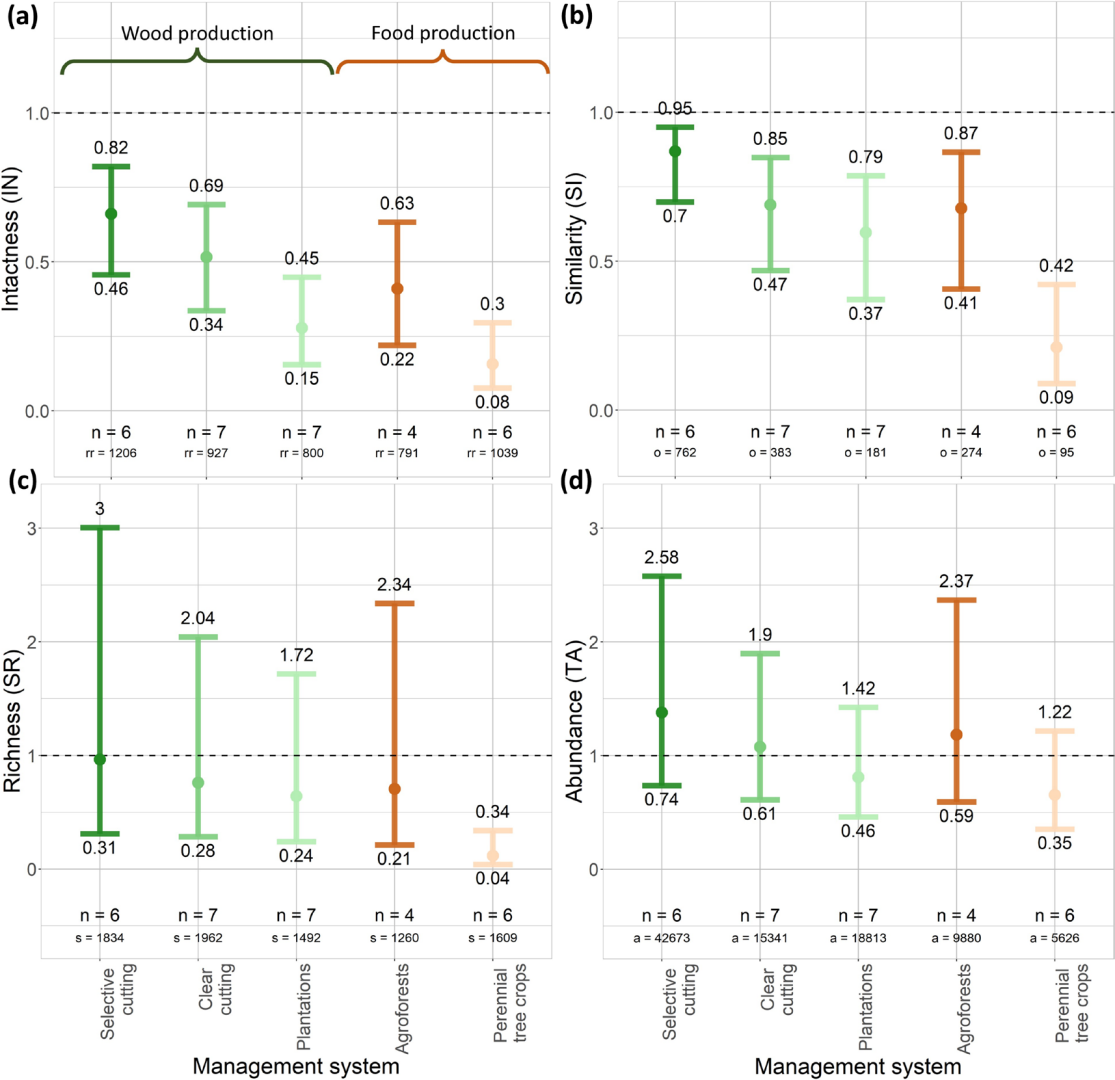
Effects of forest management on plant communities, expressed as four metrics: intactness (IN) (a), similarity (SI) (b), relative species richness (SR) (c), and relative total abundance (TA) (d). Each colored dot shows the back‐transformed mean estimate we derived from our mixed models, and the bars indicate the corresponding 95% confidence interval. The dotted line indicates the reference level (i.e., the biodiversity of the reference sites). *n* = number of studies per management type, rr = number of response values used to compute intactness, *o* = number of overlapping species between managed and reference sites, *s* = number of species occurring across the managed and reference sites, and *a* = number of abundance values used to compute relative total abundance.

Across all forest management systems, we found significantly reduced intactness of both animals (on average 23%–80% lower) and plants (34%–84% lower) (Figures 2a and 3a; Table S1). Intactness was lowest in perennial tree crops, with reductions of 80% (95% CI = 68%–88%) and 84% (70%–92%) in animal and plant intactness, respectively. We also found intactness to be considerably reduced in forest plantations, with reductions of 58% (48%–68%) and 72% (55%–85%) in animal and plant intactness, respectively. Intactness was least impaired in reduced‐impact logging systems, with reductions of 23% (7%–56%) in animal intactness. Of all animal classes, we observed the lowest intactness for herpetofauna and insects, while intactness was generally highest for mammals (Figures S3–S6; Table S1). We found a significantly higher animal intactness in agroforests than in perennial tree crop plantation systems (*p* = 0.014) (Table S2).

#### 3.2.2 Compositional Similarity

**FIGURE 4:**
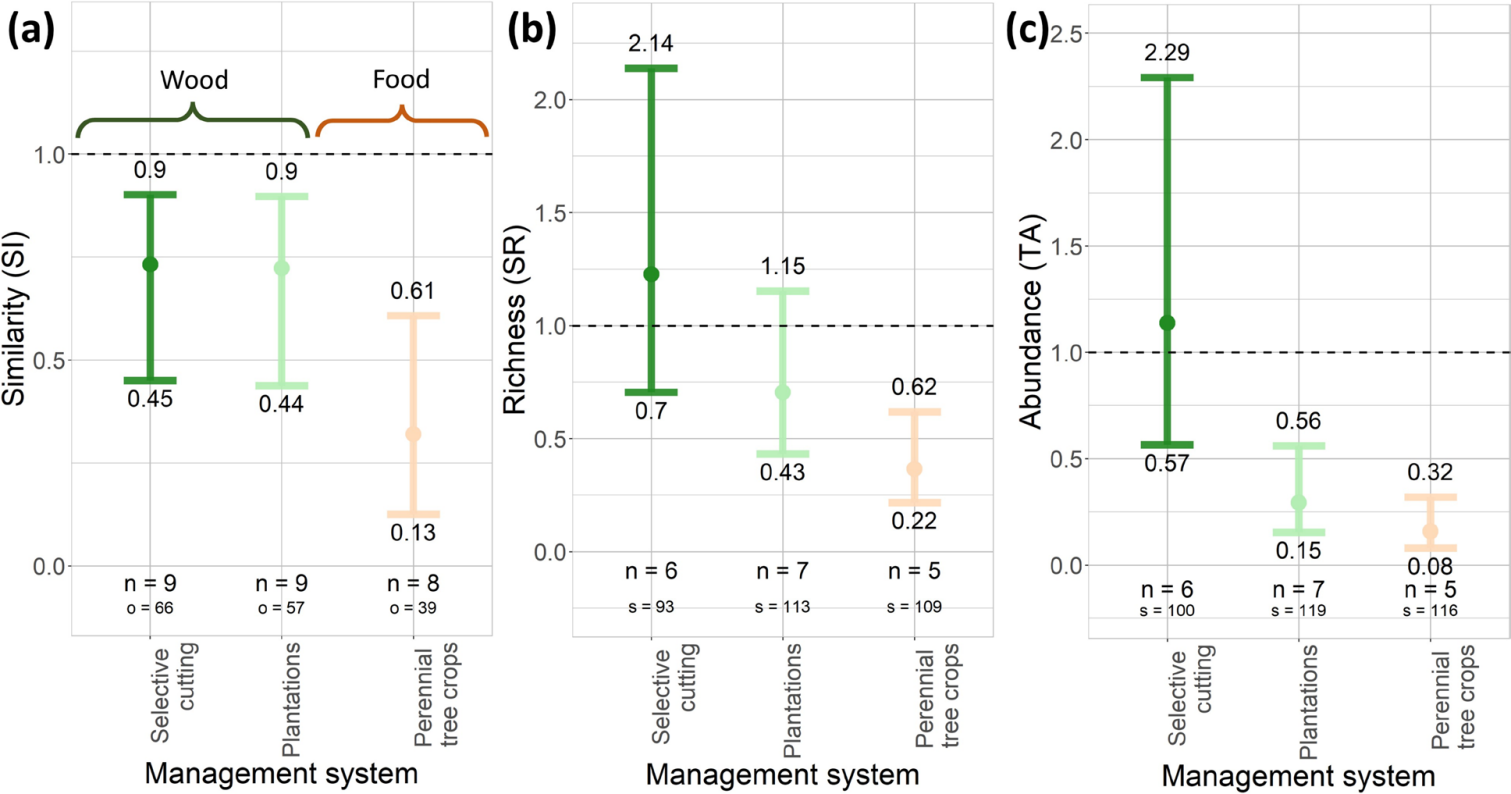
Effects of forest management on threatened animal biodiversity (i.e., insects and vertebrates), represented by species of the IUCN red list categories vulnerable to critically endangered, expressed as three metrics: similarity (SI) (a), relative species richness (SR) (b), and relative total abundance (TA) (c). Each colored dot shows the back‐transformed mean estimate we derived from our mixed models, and the bars indicate the corresponding 95% confidence interval. The dotted line indicates the reference level (i.e., the biodiversity of the reference sites). *n* = number of studies per management type, *o* = number of overlapping species between managed and reference sites, *s* = number of species occurring across the managed and reference sites, and *a* = number of abundance values used to compute relative total abundance.

For the compositional similarity metric, we found similar responses for animal and plant biodiversity across most forest management systems, with significant reductions in relative compositional similarity for both animals (on average 3%–51% lower) and plants (13%–79% lower), respectively. We found the biggest reduction in compositional similarity in perennial tree crop plantations, where compositional similarity of animals and plants was reduced by 51% (32%–69%) and 79% (58%–91%), respectively (Figures 2b and 3b; Table S1). We also observed this pattern for mammals and birds separately (Figures S3–S6; Table S1). We found a significantly reduced compositional similarity between managed and reference sites for threatened animals, ranging from 27% (10%–55%) to 68% (39%–87%) for selective cutting and perennial tree crop plantations, respectively (Figure 4a; Table S3). For wood production systems, we found a significantly lower animal compositional similarity in forest plantations than in selective cutting systems (*p* = 0.009) (Table S2). For food production systems, we found significantly higher animal compositional similarity in agroforests than in perennial tree crop plantations (*p* = 0.002).

#### 3.2.3 Relative Species Richness

We did not find significant reductions in animal relative species richness in reduced‐impact logging, selective cutting, clear cutting, and regrowth, agroforests, and silvopasture systems. For plants, we did not find significant reductions in relative species richness for selective cutting, clear cutting, and regrowth, forest plantations, and agroforest systems. For perennial tree crop and forest plantation systems, we found a reduction in animal relative species richness of 46% (34%–55%) and 25% (15%–35%), respectively (Figure 2c, Table S1). Additionally, we found an 88% (66%–96%) reduction in plant relative species richness in perennial tree crop plantations (Figure 3c; Table S1). We found a reduction in relative species richness in perennial tree crop plantations of 57% (42%–68%) for birds, 49% (1%–73%) for herpetofauna, and 41% (20%–57%) for insects (Figure S3–S6; Table S1). For forest plantations, we found a reduction in relative species richness of 39% (23%–52%) for insects and 26% (6%–41%) for mammals (Figure S3–S6; Table S1). For wood production systems, we found a significantly lower relative animal species richness in forest plantations than in selective cutting systems (*p* < 0.001) (Table S2). For food production systems, we found significantly higher relative animal species richness in agroforests than in perennial tree crop plantations (*p* = < 0.001).

#### 3.2.4 Relative Total Abundance

We did not find significant reductions in animal relative total abundance for all management systems, except for forest plantations, for which we observed a 35% (19%–47%) decline. For plants, we did not find any significant reductions in relative total abundance. We found a reduction in relative total abundance of 42% (16%–60%) for insects in forest plantations (Figures 2d and 3d; Table S1). For birds, we found a 45% (12%–66%) reduction in relative total abundance in perennial tree crop plantations (Figures S3–S6; Table S1). We also found an 84% (68%–92%) and 61% (44%–85%) reduction in relative total abundance of threatened animals in perennial tree crops and forest plantations, respectively (Figure 4c; Table S3).

### 3.3 Effects of Forest Management on Biodiversity Over Time

We found that models including time since harvesting or establishment as fixed effects explained variation in intactness significantly better than models with only the intercept for all management systems (Table S5). For compositional similarity, we found the same, except for selective cutting, for which adding time since harvesting as a fixed effect did not explain variations in similarity better than an intercept‐only model. Models with time since harvesting or establishment as a fixed effect did not explain relative species richness better than models with only the intercept. The same accounted for relative total abundance, except for perennial tree crop plantations, for which a model adding time since establishment explained variations in total abundance better than a model with only the intercept.

**FIGURE 5:**
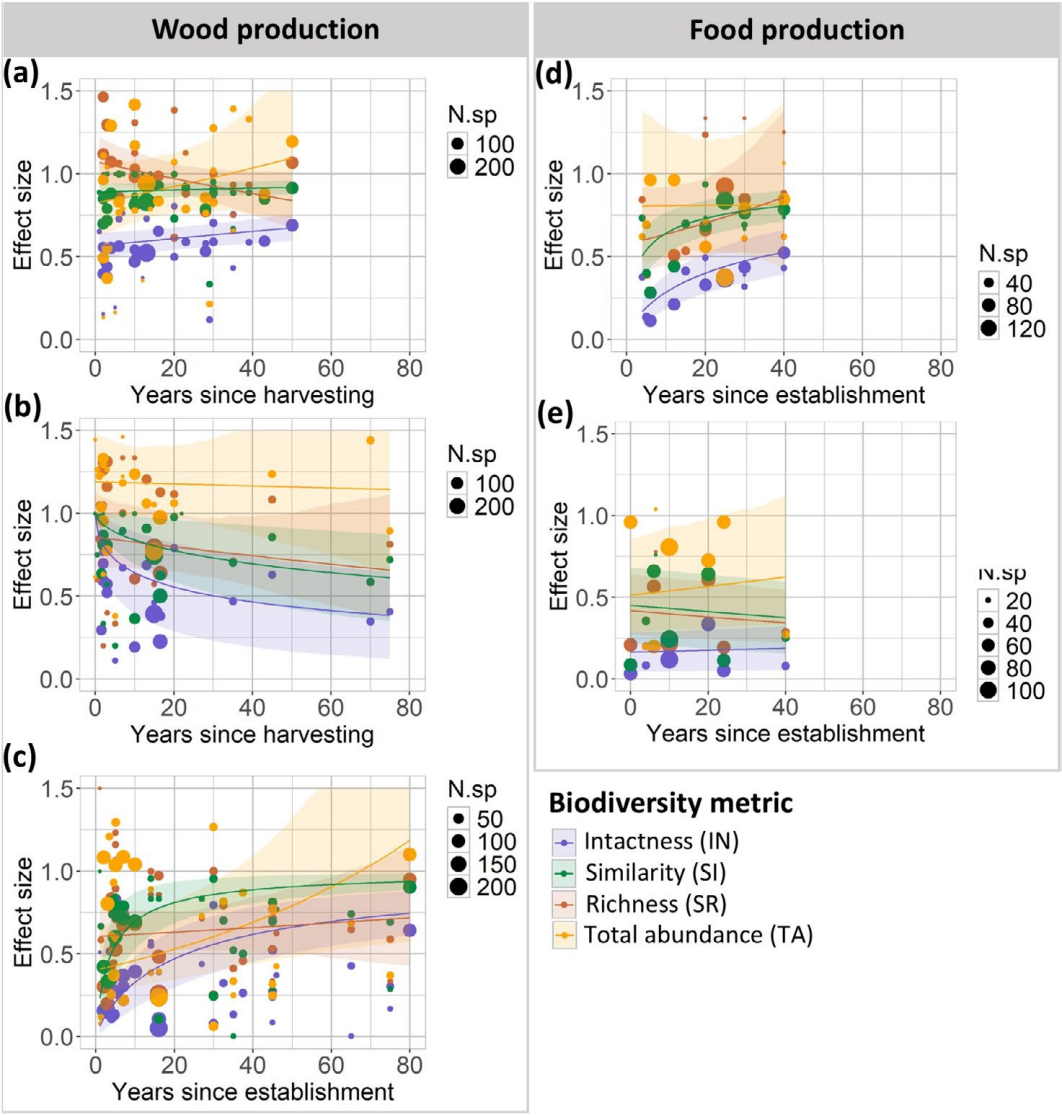
Relationships between time since tree harvesting or the establishment of a management system and animal biodiversity, expressed as intactness (IN), relative species richness (SR), proportional similarity (SI), and relative total abundance (TA) for those management systems for which enough data (≥ 5 data points) were available to build generalized linear mixed‐effect models (see Table 1 for definitions of all systems). Management systems for which enough data were available included selective cutting (a), clear cutting and natural regrowth (b), forest plantations (c), agroforests (d), and perennial tree crop plantations (e). Time since harvesting or establishment only concerns managed forests, not the reference forests. N.sp. = the number of species that are included to calculate biodiversity metrics/response ratios per time since management.

We found varying effects of forest management on animal biodiversity over time across forest management systems (Figure 5; Table S6). We found small changes in biodiversity over time in selective cutting systems; only intactness increased slightly but significantly over 50 years since tree harvesting (Figure 5a). In clear cutting systems, we observed a significant decline of ±50% in both intactness and compositional similarity over a period of 70 years (Figure 5b). In forest plantation systems, we found a logarithmic increase in intactness and compositional similarity of ±70% and ±80%, respectively, over a period of 80 years after establishment (Figure 5c). We found a significant ±30% increase in both intactness and compositional similarity in agroforestry systems over a period of 40 years after establishment (Figure 5d). For perennial tree crops, we found both positive and negative changes in biodiversity over time; we observed slight but significant increases of ±5% in intactness and ±10% in relative total abundance, and slight but significant declines of ±10% in compositional similarity over a period of 40 years since establishment (Figure 5e).

### 3.4 Effects of Taxonomic Class and Continent

We found that models with taxonomic class as an additional fixed effect besides management systems outperformed those with only management systems for intactness and compositional similarity but not for relative species richness and total abundance (Table S7). We observed no significant improvement in the predictive power of animal biodiversity models when adding continent as an additional fixed effect besides the management systems (Table S7).

## 4 Discussion

We conducted a global assessment of the effects of seven forest management systems on biodiversity over time, captured in four distinct biodiversity metrics (see Table 2 and Table S8 for a summary of the results). We found overall larger effects of forest management on intactness and compositional similarity than on relative species richness and total abundance. For wood production systems, we found smaller negative effects of selective and clear cutting and natural regrowth than of forest plantations on biodiversity, relative to undisturbed forest reference sites. For food production systems, we showed smaller negative effects of agroforests and silvopasture than of perennial tree crop plantations on biodiversity, relative to undisturbed forests. We observed substantial increases in intactness and similarity in agroforest and forest plantations over time since establishment, but observed limited significant changes in richness and abundance.

### 4.1 Effects of Forest Management on Biodiversity

For both wood and food production systems, we showed that the magnitude of the effects of forest management on biodiversity depends on the metric in which biodiversity is captured. We found that the magnitude of effects was largest for intactness, followed by compositional similarity, species richness, and total abundance. Similar relative species richness and total abundance, in combination with a reduction of intactness and compositional similarity across management systems, indicates that a loss of species in the managed sites is often compensated for by an influx of species that do not occur in the reference sites. This finding highlights the need to use a combination of intactness, similarity, and richness metrics to identify the potential of managed forests to host species specifically adapted to undisturbed forests, as well as the potential of managed forests to host species from other areas. For instance, if only relative species richness is considered, the effects of management on specialist species or changes in the types and abundances of species may go unnoticed. In contrast, if only intactness is considered, an influx of species that do not occur in reference sites may remain unobserved. Using a combination of intactness, similarity, and richness metrics is particularly relevant when the effects of forest management on biodiversity are assessed for taxonomic groups that are overall least affected by management, such as mammals (Chaudhary et al. 2016). Using only relative species richness may suffice when specifically studying taxonomic groups that strongly respond to forest management and for which strong declines in species richness in response to forest management have consistently been observed in prior studies, such as for saproxylic beetles (Müller et al. 2008) and bryophytes (Király et al. 2013).

Besides differences in the magnitude of the effects of forest management on biodiversity across biodiversity metrics, our findings suggest that some taxonomic groups respond more strongly to forest management than others. For example, we showed a generally larger impact of management on intactness for herpetofauna and insects than for bird and mammal classes, in line with previous studies (Chaudhary et al. 2016; López‐Bedoya et al. 2022). This may be explained by stronger responses of herpetofauna and insect species than bird and mammal species to changes in forest structure and composition. The stronger response of herpetofauna to forest management might also be explained by a loss of aquatic habitat when forests are harvested or plantations established (Warrington et al. 2017). Despite differences found in the effects of forest management on biodiversity across taxonomic groups, the impacts of management on species richness across taxonomic classes were generally smaller than those found in other studies (Chaudhary et al. 2016; Paillet et al. 2010). This may be explained by differences in the taxonomic groups studied by others and herein. Other studies often assess other types of taxonomic groups, such as fungi and lichens, that were not considered in this study. Additionally, effects of forest management on specific vulnerable species within taxonomic groups, such as saproxylic beetles and bryophytes, may have been overshadowed by grouping animal species into broader taxonomic groups in our study (Paillet et al. 2010).

Despite differences in responses to forest management between biodiversity metrics and taxonomic groups, we observed various generalities. We found mostly larger effects of intensive than of extensive management on biodiversity. Within wood production systems, we found that extensively managed forests (e.g., selective cutting systems) have a higher biodiversity than intensively managed forests (e.g., forest plantations). This difference is likely related to the more modest effect that selective harvesting of trees tends to have on forest vegetation structure and heterogeneity (Addo‐fordjour et al. 2022; Robinson and Robinson 1999; Von Oheimb and Härdtle 2009), compared to forest plantations, in which the planting of a single or very few, often non‐native, tree species results in a homogeneous vegetation structure (Chazdon and Guariguata 2016). The homogeneous vegetation structure and diversity of forest plantations may also explain the significantly lower total abundance of threatened animal species found in forest plantations than in reference sites, indicating that forest plantation expansion on natural forest land could further endanger species.

Similarly, we found generally larger effects of intensive (e.g., perennial tree crops) than of extensive food production (e.g., agroforests) on biodiversity, relative to reference sites. Relatively small differences in biodiversity between reference sites and agroforests are likely related to the presence of multiple layers in the canopy and multiple tree and shrub/crop species that often characterize agroforests and which may provide habitat for a range of different species, including those adapted to undisturbed forests (Bohada‐Murillo et al. 2020; Schroth et al. 2004; Torralba et al. 2016). In contrast, we found significantly lower biodiversity in perennial tree crop plantations than in reference sites across taxonomic groups and biodiversity metrics. We also found significantly lower intactness, compositional similarity, and relative species richness in perennial tree crop plantations than in agroforests, which corroborates prior findings (Savilaakso et al. 2014; Yahya et al. 2022). Finally, we showed a significantly lower species richness and total abundance of threatened animal species in perennial tree crop plantations than in reference sites. Altogether these findings reveal that the introduction of perennial tree crop plantations has a profoundly negative effect on many different aspects of biodiversity, which is likely related to the introduction of, often, non‐native tree species in monoculture, which results in a homogeneous vegetation structure, including a single‐storied canopy (Fitzherbert et al. 2008).

### 4.2 Effects of Forest Management on Biodiversity Over Time

Our results suggest that the effects of forest management on biodiversity may change over time after tree harvesting or since the establishment of management. In forest plantations, we observed substantial and rapid increases in intactness and similarity over time since establishment, while we found no significant change in relative species richness and total abundance. This indicates that animal compositions of forest plantations become increasingly similar to those of undisturbed forests over time. Temporal increases in intactness and similarity may be related to an increasingly more similar vegetation structure and composition to that of undisturbed forests over time, which provides suitable habitats for an increasing number of species that occur in undisturbed forests (Hua et al. 2022; Tudge et al. 2023). The results suggest a benefit of introducing longer rotation periods in forest plantations, so as to increase habitats for species specifically adapted to undisturbed forests. Additionally, our results suggest that changes in alpha diversity over time in forest plantations could influence the heterogeneity of species compositions and assemblages across the landscape in areas with a heterogeneous mix of patches of plantation forests that are established at different moments in time.

In contrast to forest plantations, we showed only small increases in intactness over 50 years after selective cutting. This indicates that, although selective cutting has a relatively small effect on biodiversity compared to other management systems, effects remain persistent for decades. This corroborates previous findings (Crouzeilles et al. 2016) and may relate to persistent changes in forest structure (Gatti et al. 2015). The same may hold for clear cutting and natural regrowth systems, for which, in contrast to our expectations, we found a decline in intactness and compositional similarity over a period of 70 years after tree harvesting. This contrasts with prior studies that show increases in biodiversity over time after clear cutting as a result of increased heterogeneity in forest structure (Taki et al. 2013; Xu et al. 2015). Our results may be explained by studies that find that the number of species across taxonomic groups is high in early successional stages, after which it first declines throughout mid‐successional stages and then rises again during late‐successional stages (Hilmers et al. 2018; Swanson et al. 2011). As the database included limited biodiversity data obtained longer than 20 years after clear‐cut harvesting, our results may only reflect the expected decline in biodiversity upon a transition from early‐successional to mid‐successional stages and not the expected rise in biodiversity during late‐successional stages. Additionally, we may have observed an extinction debt, under which species may still be present in the first years following clear cutting, after which they gradually disappear over time (Wearn et al. 2012).

In agroforests, we found strong increases in intactness and compositional similarity in the years following establishment. In combination with limited changes in relative species richness and total abundance over time, the increases in intactness and compositional similarity indicate that animal communities of agroforests become more similar to those of undisturbed forests. This could indicate that the vegetation structure and composition of agroforests becomes more complex over time and more alike that of undisturbed forests as a result of tree and shrub growth and changes in their composition (De Leijster et al. 2021), despite the continued production of crops underneath the canopy and potential occasional cutting and planting of trees. This finding highlights the potential of agroforests to mitigate biodiversity loss in agricultural areas and may explain why previous studies capture such variable effects of agroforests on biodiversity. Prior studies may have measured biodiversity at different times since agroforest establishment (Torralba et al. 2016) or may have used different metrics to capture biodiversity changes over time, which, as we observed in this study, capture varying responses of different aspects of biodiversity to forest management. In contrast to agroforests, we showed limited and contrasting changes in biodiversity in perennial tree crop plantations over 40 years since establishment. This finding may reflect a lack of vegetation succession and frequent disturbances as a result of crop harvesting in perennial tree crop plantations and corroborates studies that show limited recovery of biodiversity in perennial tree crop plantations (e.g., in oil palm plantations) (Ashton‐Butt et al. 2019).

### 4.3 The Robustness of Our Findings

Although we compiled a large database, biases in the data and data limitations could have affected our biodiversity estimates and the biodiversity responses to forest management over time we observed. For instance, more data were available on animals than on plants, and less data were available on reduced‐impact logging and silvopasture systems than on selective cutting and forest plantation systems. This makes the estimates of plants less robust than those of animals, and the estimates for reduced‐impact logging and silvopasture systems less robust than those for selective cutting and forest plantation systems. Additionally, data availability was skewed toward years close to harvesting or establishment. For instance, most studies on forest plantations were conducted within the first two decades since plantation establishment. This may have resulted in relatively low overall biodiversity estimates for plantations, indicating the importance of considering the potential for biodiversity recovery in this system. Skewness in data availability toward years close to harvesting or establishment may explain unexpected responses of biodiversity over time across management systems, such as reductions of intactness and compositional similarity over time following clear cutting and regrowth. Additionally, we may have failed to capture the effects of important ecological processes on biodiversity changes, such as the decline and subsequent recovery of biodiversity along successional stages (Hilmers et al. 2018). Extinction debt processes, during which species slowly disappear over time due to a lagged response to disturbances, may have further influenced the trajectory of change in biodiversity over time we observed after harvesting or establishment.

Our results were likely influenced by variations in management intensity and management approaches adopted within each individual management system. There can be substantial variations in wood harvesting intensity (e.g., the number of trees cut per unit area) and food production intensity (e.g., use of pesticides and herbicides) within individual management systems around the world (Hari Poudyal et al. 2018). For example, high‐intensity selective cutting may affect biodiversity more than low‐intensity selective cutting (França et al. 2024). Additionally, variations in management practices, such as the planting of native versus non‐native tree species in forest plantations, could produce varying biodiversity responses. Biases in data availability toward the tropics could have prevented us from capturing the complete range of wood and food production intensities across the world. For instance, caution should be taken when interpreting our results for agroforests, as most data in these systems were sampled in tropical regions, where agroforests are known to have a relatively heterogeneous vegetation structure and composition (Schroth et al. 2004). Additionally, fewer pesticides and herbicides may be used in agroforests in tropical regions than those common in temperate regions (Tang et al. 2021). Finally, the effects of other drivers of biodiversity loss that have not been considered in this study, such as pollution and climate change, may have added further variability in our data that we have not accounted for in this study.

Adding continents as additional fixed effects in models besides management and restoration systems did not improve our animal biodiversity models, indicating that our results are generalizable across space for animal biodiversity. Yet, it is important to note that we did not assess the effects of forest management on all taxonomic classes (e.g., fungi and non‐insect arthropods were omitted). These unaddressed taxonomic classes may respond differently to forest management than the classes included in this study. Additionally, our data may have been biased toward species groups that are generally less affected by forest management, such as mammals, hereby omitting more sensitive species groups, such as saproxylic beetles and fungi (Chaudhary et al. 2016; Paillet et al. 2010). Such biases in the data potentially explain the proportionally smaller effects of forest management on relative species richness we found compared to prior studies (Chaudhary et al. 2016; Paillet et al. 2010). Besides this, the effects of forest management on biodiversity over time may vary across taxonomic classes (Romanelli et al. 2022). We were unable to test the interaction between time since harvesting or establishment and taxonomic class due to data limitations. Future studies may explore such interactions to unravel differences across taxonomic groups in the effects of forest management on biodiversity over time. Finally, it is important to recognize that we assessed the effects of forest management on alpha diversity without considering the configuration of the surrounding landscape (e.g., heterogeneity of land uses), even though this can have profound modulating effects on local biodiversity responses (Batáry et al. 2011; Uhl et al. 2024).

## 5 Conclusion

We provide a global assessment of the effects of forest management on alpha animal and plant biodiversity, relative to the biodiversity of reference sites (i.e., undisturbed forests). We used mixed‐effects modeling to estimate the impacts of seven forest management systems on four biodiversity metrics based on data from three global biodiversity databases. Overall, we found larger differences in intactness and compositional similarity than in species richness and total abundance between managed and undisturbed forests. This indicates that a decline in species specifically adapted to undisturbed forests occurs upon a transition to forest management, but that this loss is partially offset by an influx of species from elsewhere. This finding highlights the need to use a combination of intactness, compositional similarity, and relative species richness metrics to identify the potential of managed forests to host species specifically adapted to undisturbed forests, as well as the potential of managed forests to host species from elsewhere. When considering differences in biodiversity effects across systems, we found that extensive forest management (e.g., selective cutting and agroforests) generally has lower impacts on biodiversity than intensive forest management (e.g., forest and perennial tree crop plantations). Additionally, we found that both intactness and similarity increase significantly over time in forest plantations and agroforests, while relative species richness and total abundance remain stable. This indicates that animal species compositions of forest plantations and agroforests can become increasingly similar to those of undisturbed forests over time. Therefore, our results highlight the potential of transitions from intensive to extensive forest management to mitigate biodiversity loss related to wood and food production. At the same time, our results highlight the benefit of introducing longer rotation periods in forest plantations and fostering long‐term transitions to agroforests on non‐forest land to increase habitats for species specifically adapted to undisturbed forests. In the future, our findings could be integrated in global biodiversity models to run scenarios aimed at better understanding how different forest management systems may be combined and applied over space and time to bend the curve of biodiversity loss.

## Author Contributions

**Hanneke van 't Veen:** conceptualization, data curation, formal analysis, investigation, methodology, validation, visualization, writing – original draft, writing – review and editing. **Koen Kuipers:** data curation, investigation, methodology, validation, writing – review and editing. **Aafke Schipper:** investigation, methodology, validation, writing – review and editing. **Alexandra Marques:** investigation, methodology, supervision, validation, writing – review and editing. **Mart‐Jan Schelhaas:** investigation, methodology, validation, writing – review and editing. **Rob Alkemade:** conceptualization, funding acquisition, investigation, methodology, resources, supervision, writing – review and editing.

## Conflicts of Interest

The authors declare no conflicts of interest.

## Supporting information


Appendix S1.

## Data Availability

The data and code that support the findings of this study are openly available in Zenodo at https://doi.org/10.5281/zenodo.15276783. Biodiversity data were obtained from the PREDICTS 2016 database via the Natural History Museum at https://doi.org/10.5519/0066354, from the database of Kuipers et al. (2023) available on Zenodo at http://doi.org/10.5281/zenodo.8199982, from the GLOBIO database available on Zenodo at https://doi.org/10.5281/zenodo.15528625, and from the IUCN Red List of Threatened Species available at https://www.iucnredlist.org.
