# Supplementary material for: A Global Assessment of Plant and Animal Community Responses to Forest Management Over Time
Source: Glob Chang Biol. 2025 Jun 16;31(6):e70279. doi: 10.1111/gcb.70279 (PMC12169101; doi:10.1111/gcb.70279)
Supplement: Supplementary file 1 — Appendix S1. [file GCB-31-e70279-s001.zip › gcb70279-sup-0001-Appendix/gcb70279-sup-0002-TableS8.pdf]

Supplementary information

Table S8. Hypothesized effects of forest management on animal and plant biodiversity, general findings and whether they are in line with our hypotheses or not. We highlighted expected differences in biodiversity responses to forest management across taxonomic groups and biodiversity metrics. We based our hypotheses on prior, often local scale, research on the response of biodiversity to changes in vegetation structure and composition. The color coding matches that of Table 1 and the effect size figures of the main paper.

	Management type	Vegetation structure and composition	Hypothesized effects on biodiversity	General findings on effects of management on biodiversity	In line with hypotheses
Predominantly wood production	Reduced-impact logging (RI)	• Heterogeneous vegetation structure<br>• Full-storied or multi-layered canopy.<br>• Multiple tree species<br>• Native tree species unless invaded by non-native species<br>• Scattered gaps in the canopy, which are smaller in size as those under SC	We hypothesized that biodiversity is only slightly lower in RI than reference sites because of the, by design, small effect of RI on vegetation structure and composition (Bicknell, Struebig, and Davies 2015; Schwartz et al. 2012). We expected the selective harvesting of trees under RI to slightly affect tree diversity and composition (Schwartz et al. 2012), which we hypothesized to negatively influence intactness and similarity across taxonomic groups. Yet, we expected the presence of small gaps in the canopy to enhance bird richness and abundance (Przepióra, Loch, and Ciach 2020). In contrast, we expected neutral (Gitzen and West 2002) or negative (Cudney-Valenzuela et al. 2023) effects of canopy gaps on mammal richness.	We found slight, yet significantly lower animal intactness and similarity in RI than in reference sites across taxonomic groups. We found similar richness and total abundance in RI as in reference sites across taxonomic groups.	Partially. A slightly lower intactness and similarity in RI than in reference sites was expected, as were smaller relative effects of RI on richness and total abundance compared to intactness and similarity. We did not observe the expected increase in bird richness and abundance in response to gaps in the forest canopy.
	Selective cutting (SC)	• Heterogeneous vegetation structure<br>• Full-storied or multi-layered canopy<br>• Multiple tree species<br>• Native tree species unless invaded by non-native species<br>• Scattered gaps, which are bigger in size as those under RI	We hypothesized that, relative to reference sites, SC generally has larger effects on vegetation structure and composition than RI (Imai et al. 2012), as generally more trees are cut under selective logging and less attention is paid to minimizing effects of logging on biodiversity (Pereira et al. 2002). Hence, we expected larger differences between the biodiversity SI and reference sites than found for RI across biodiversity metrics. We expected the selective harvesting of trees under SC to significantly affect tree diversity and composition (Imai et al. 2012), as well as the vertical structure of the canopy (Okunda et al. 2003), which we hypothesized to negatively influence biodiversity across metrics and taxonomic groups. Yet, like for RI, we expected the presence of gaps in the	We showed slight, yet significantly, lower intactness and similarity in SC than in reference sites across taxonomic groups. We found similar richness and total abundance in SC as in reference sites across taxonomic groups. We showed a slight increase in intactness over a period of 50 years after logging.	Partially. A slightly lower biodiversity in SC than in reference sites was expected, as were smaller relative effects on richness and total abundance compared to intactness and similarity. We observed slightly higher estimates of animal richness than reference site richness, yet the confidence interval overlapped with reference richness. We did not observe expected increases in insect, bird, reptile and plant richness in

			canopy to positively influence insect (Perry et al. 2018), bird (Przepióra, Loch, and Ciach 2020), reptile (Greenberg 2001) and plant (Duguid et al. 2013) species richness and abundance. In contrast, we expected neutral (Gitzen and West 2002) or negative (Cudney-Valenzuela et al. 2023) effects of canopy gaps on mammal richness and abundance, as well as neutral effects on amphibian richness and abundance (Greenberg 2001). We expected slow recovery of biodiversity over time under SC (Gatti et al. 2015).		response to gaps in the forest. In line with our expectation, we showed a slow increase in intactness over time under AG. Yet, we did not observe expected increases in richness, similarity and total abundance over time. Additionally, we found similar biodiversity between SC and RI systems.
	Clear cutting and regrowth (CC)	• Homogeneous vegetation structure<br>• Single-storied canopy<br>• Multiple tree species<br>• Native tree species, unless invaded by non-native species<br>• No gaps in the canopy	We hypothesized that CC has significantly lower biodiversity than reference sites across taxonomic groups and across metrics because of its homogeneous vegetation structure, including the single-storied canopy (Chaudhary et al. 2016; Crouzeilles et al. 2016). Because of the homogeneous vegetation structure and single-storied canopy of CC, we expected its effect on biodiversity to be significantly more negative than that of SC and RI (Xu et al. 2015). We expected larger effects of CC on intactness and similarity than on species richness and abundance, as we expected an influx of species (e.g., early successional or generalist species) that do not occur in reference sites, and increases in abundances of some species and declines in others (Bogdziewicz and Zwolak 2014). We hypothesized animal biodiversity to recover over time following natural forest regrowth along successional gradients (Xu et al. 2015); we expected faster recovery of richness and abundance and slower recovery of intactness and similarity.	We found significantly lower intactness and similarity in CC than in reference sites across taxonomic groups. Relative to reference sites, we found relatively low intactness for herpetofauna, and plants compared to other taxonomic groups. We found similar richness and total abundance in CC as in reference sites across taxonomic groups, except for plants for which we observed significantly lower richness in CC than in reference sites. We showed a decline in intactness and similarity over 70 years after harvesting.	Partially. A lower overall biodiversity in CC than in reference sites was expected, as were smaller relative effects on richness and total abundance compared to intactness and similarity. Yet, we expected significantly lower richness and total abundance in CC than in reference sites. Additionally, we expected a significantly lower biodiversity in CC than in SC and RI. Finally, we expected biodiversity to increase over time upon forest regrowth, not decline.
	Forest plantations (PL)	• Homogeneous vegetation structure<br>• Single-storied canopy	We hypothesized that PL has significantly lower biodiversity than reference sites across taxonomic groups and biodiversity metrics because of its homogeneous vegetation structure, including its single-storied canopy and generally low numbers of tree species	We showed significantly lower overall animal and plant biodiversity across biodiversity metrics compared to reference sites. We found similar richness and total abundance to that of	Partially. A lower overall biodiversity in PL than in reference sites was expected, as was a significantly lower richness and similarity in PL than SC

		• Mostly single tree species<br>• Planted tree species are either non-native or native<br>• No gaps in the canopy	(Chaudhary et al. 2016; Crouzeilles et al. 2016). We expected significantly larger effects of PL on biodiversity than of CC, relative to reference sites, because we expected a lower tree diversity and a higher chance that non-native tree species are planted, which affect biodiversity more than native tree species (Wang et al. 2022). Like for CC, we expected significantly lower biodiversity in PL than in SC and RI systems. Finally, we expected animal biodiversity to increase slightly with plantation age across taxonomic groups (Tudge et al. 2023) as a result of enhanced tree heights, slight increases in the heterogeneity of vegetation structure and an expected influx of tree species and understory vegetation over time (Eycott, Watkinson, and Dolman 2006).	reference sites for birds and herpetofauna, as well as similar richness for mammals as in reference sites. We found strong significant increases of ± 0.7 and ± 0.25 in intactness and similarity over an 80-year period since PL establishment.	and PL. Yet, we expected to find significantly lower biodiversity in PL than in reference sites across all taxonomic groups examined across all metrics. Although we hypothesized to find a small increase in biodiversity over time, we did not expect intactness and similarity to increase at a relatively fast rate in PL systems.
Predominantly food production	Agroforests(AG)	• Heterogeneous vegetation structure<br>• Two-storied or multi-layered canopy<br>• Multiple tree species<br>• Native, if established in natural forests, unless invaded by non-native species. Non-native or native trees are planted if established in non-forested land<br>• Scattered gaps in the canopy	We hypothesized to find lower biodiversity in AG than reference sites because we expect its vegetation structure, including canopy layering, to be less heterogeneous than that of reference sites and its tree composition less diverse (Perry et al. 2016). We expected that AG affects intactness and similarity more than richness and total abundance, as we expected an influx of species that do not occur in reference sites and increases in abundances of some species and declines in others (Jose 2012; Harvey and González Villalobos 2007). Besides this, we expected that planting, growing and harvesting of crops affects biodiversity overall negatively, in particular mammal biodiversity, because we expected this taxonomic group to avoid human presence (Ferreira et al. 2020), as well as insects and plants because of expected use of pesticides and herbicides. Yet, we also expected that the presence of gaps in the canopy may positively influence insect (Perry et al. 2018), bird (Przepióra, Loch, and Ciach 2020), reptile (Greenberg 2001) and plant (Duguid et al. 2013) species richness and abundance, which may compensate for the negative effects of crop growth and harvesting. In contrast, we expected	We found significantly lower intactness and similarity in AG than in reference sites across taxonomic groups. We found similar richness and total abundance in AG as in reference sites across taxonomic groups. We observed strong significant increases of ± 0.25 in intactness and similarity over a 40-year period since AG establishment.	Partially. A lower overall biodiversity in AG than in reference sites was expected, as were smaller relative effects of AG on richness and total abundance compared to intactness and similarity. Yet, we expected to find larger differences between the biodiversity of AG systems and reference sites. Additionally, we did not expect intactness and similarity to increase at a relatively fast rate in AG systems. We did not find expected larger effects of AG on mammal, insect and plant biodiversity compared to other taxonomic groups across biodiversity metrics.

			neutral (Gitzen and West 2002) or negative (Cudney-Valenzuela et al. 2023) effects of canopy gaps on mammal richness and abundance, as well as neutral effects of canopy gaps on amphibian richness and abundance (Greenberg 2001). We expected a slight increase in animal biodiversity over time since establishment as trees age (De Leijster et al. 2021).		
	Silvopasture (SP)	• Heterogeneous vegetation structure<br>• Single-storied or multi-layered canopy<br>• Multiple tree species<br>• Native, if established in natural forests, unless invaded by non-native species. Non-native or native trees are planted if established in non-forested land<br>• Scattered gaps in the canopy	We hypothesized to find lower biodiversity in SP than reference sites because we expected its vegetation structure, including canopy layering, to be less heterogeneous than that of reference sites and its tree composition less diverse (Orefice et al. 2017). We expected that SP affects intactness and similarity more than richness and total abundance, as we expected an influx of species that do not occur in reference sites and increases in abundances of some species and declines in others (Perez-Alvarez et al. 2023; Orefice et al. 2017). We hypothesized SP to affect biodiversity slightly more than AG, relative to reference sites, because we expected more heterogeneity in canopy layers in AG, as a result of crop production. We expected that the presence of gaps in the canopy positively influence insect (Perry et al. 2018), bird (Przepióra, Loch, and Ciach 2020), reptile (Greenberg 2001) and plant (Duguid et al. 2013) species richness and abundance, which may compensate for negative effects of livestock production. In contrast, we expected neutral (Gitzen and West 2002) or negative (Cudney-Valenzuela et al. 2023) effects of canopy gaps on mammal richness and abundance, as well as neutral effects of gaps in the canopy on amphibian richness and abundance (Greenberg 2001).	We found significantly lower intactness and similarity in SP than in reference sites across taxonomic groups. We found similar richness and total abundance in SP as in reference sites across taxonomic groups.	Partially. A lower overall biodiversity in SP than in reference sites was expected, as were smaller relative effects of SP on richness and total abundance compared to intactness and similarity. Yet, we expected to find larger differences between the biodiversity of SP systems and reference sites. We did not find the expected larger effects of SP on mammal, insect and plant biodiversity compared to other taxonomic groups across biodiversity metrics.
	Perennial tree crops (PC)	• Homogeneous vegetation structure	We hypothesized to find significantly lower biodiversity in PC than reference sites across taxonomic groups and biodiversity metrics because of its homogeneous vegetation	We showed significantly lower intactness and similarity under PC than in reference sites across taxonomic groups. We	Partially. A significantly lower overall animal and plant intactness, similarity and abundance in PC than

		• Single-storied canopy<br>• Mostly single tree species<br>• Planted tree species are either non-native or native<br>• No gaps in the canopy	structure, including its single-storied canopy and generally low numbers of tree species (Savilaakso et al. 2014). We expected significantly larger effects of PC than of AG and SP on biodiversity (Yahya et al. 2022), because of its more homogeneous vegetation structure, lower tree diversity, as well as a higher chance that non-native tree species are planted, which affect biodiversity more than native tree species (Wang et al. 2022). Besides this, we hypothesized that the planting, growing and harvesting of crops to affect biodiversity, in particular mammal biodiversity, because we expected this taxonomic group to avoid human presence (Ferreira et al. 2020), as well as insects and plants because of expected use of pesticides and herbicides. We did not expect increases in biodiversity over time because we expect that the vegetation structure and tree composition of PC to remain similar as a result of continuous management activities, such as weeding, thinning and the application of pesticides and herbicides.	found the same for animal richness overall, as well as birds, herpetofauna and insect richness. Yet, for plants and mammals, we found similar richness in PC as in reference sites and total abundance was only significantly lower than reference sites for birds. We found significantly lower intactness, similarity and richness in PC than in AG. We found limited and contrasting changes in biodiversity over a 40-year period for this system.	in reference sites was expected, as well as the limited change in biodiversity over time. Yet, we did not find the expected significant differences in biodiversity between PC and reference sites across all taxonomic groups and metrics. Additionally, we did not find expected significant differences between the biodiversity of PC and SP.

References

Bicknell, Jake E., Matthew J. Struebig, and Zoe G. Davies. 2015. “Reconciling Timber Extraction with Biodiversity Conservation in Tropical Forests Using Reduced-Impact Logging.” *Journal of Applied Ecology* 52 (2): 379–88. https://doi.org/10.1111/1365-2664.12391.

Bogdziewicz, Michał, and Rafał Zwolak. 2014. “Responses of Small Mammals to Clear-Cutting in Temperate and Boreal Forests of Europe: A Meta-Analysis and Review.” *European Journal of Forest Research* 133 (1): 1–11. https://doi.org/10.1007/s10342-013-0726-x.

Chaudhary, A., Z. Burivalova, L. P. Koh, and S. Hellweg. 2016. “Impact of Forest Management on Species Richness: Global Meta-Analysis and Economic Trade-Offs.” *Scientific Reports* 6: 1–10. https://doi.org/10.1038/srep23954.

Crouzeilles, Renato, Michael Curran, Mariana S. Ferreira, David B. Lindenmayer, Carlos E.V. Grelle, and José M. Rey Benayas. 2016. “A Global Meta-Analysis on the Ecological Drivers of Forest Restoration Success.” *Nature Communications* 7 (May): 1–8. https://doi.org/10.1038/ncomms11666.

Cudney-Valenzuela, Sabine J., Víctor Arroyo-Rodríguez, José C. Morante-Filho, Tarin Toledo-Aceves, and Ellen Andresen. 2023. “Tropical Forest Loss Impoverishes Arboreal Mammal Assemblages by Increasing Tree Canopy Openness.” *Ecological Applications* 33 (1): 1–12. https://doi.org/10.1002/eap.2744.

Duguid, M C, B R Frey, D S Ellum, M Kelty, and M S Ashton. 2013. “The Influence of Ground Disturbance and Gap Position on Understory Plant Diversity in Upland Forests of Southern New England.” *Forest Ecology and Management* 303: 148–59. https://doi.org/10.1016/j.foreco.2013.04.018.

Eycott, A. E., A. R. Watkinson, and P. M. Dolman. 2006. “Ecological Patterns of Plant Diversity in a Plantation Forest Managed by Clearfelling.” *Journal of Applied Ecology* 43 (6): 1160–71. https://doi.org/10.1111/j.1365-2664.2006.01235.x.

Ferreira, Aluane Silva, Carlos A. Peres, Pavel Dodonov, and Camila Righetto Cassano. 2020. “Multi-Scale Mammal Responses to Agroforestry Landscapes in the Brazilian Atlantic Forest: The Conservation Value of Forest and Traditional Shade Plantations.” *Agroforestry Systems* 94 (6): 2331–41. https://doi.org/10.1007/s10457-020-00553-y.

Gatti, R. C., S. Castaldi, J. A. Lindsell, D. A. Coomes, M. Marchetti, M. Maesano, A. Di Paola, F. Paparella, and R. Valentini. 2015. “The Impact of Selective Logging and Clearcutting on Forest Structure, Tree Diversity and above-Ground Biomass of African Tropical Forests.” *Ecological Research* 30 (1): 119–32. https://doi.org/10.1007/s11284-014-1217-3.

Gitzen, Robert A., and Stephen D. West. 2002. “Small Mammal Response to Experimental Canopy Gaps in the Southern Washington Cascades.” *Forest Ecology and Management* 168 (1–3): 187–99. https://doi.org/10.1016/S0378-1127(01)00745-9.

Greenberg, Cathryn H. 2001. “Response of Reptile and Amphibian Communities to Canopy Gaps Created by Wind Disturbance in the Southern Appalachians.” *Forest Ecology and Management* 148 (1–3): 135–44. https://doi.org/10.1016/S0378-1127(00)00486-2.

Harvey, Celia A., and Jorge A. González Villalobos. 2007. “Agroforestry Systems Conserve Species-Rich but Modified Assemblages of Tropical Birds and Bats.” *Biodiversity and Conservation* 16 (8): 2257–92. https://doi.org/10.1007/s10531-007-9194-2.

Imai, N., T. Seino, S. Aiba, M. Takyu, J. Titin, and K. Kitayama. 2012. “Effects of Selective Logging on Tree Species Diversity and Composition of Bornean Tropical Rain Forests at Different Spatial Scales.” *Plant Ecology* 213 (9): 1413–24. https://doi.org/10.1007/s11258-012-0100-y.

Jose, Shibu. 2012. “Agroforestry for Conserving and Enhancing Biodiversity.” *Agroforestry Systems* 85 (1): 1–8. https://doi.org/10.1007/s10457-012-9517-5.

Leijster, V. De, M. J. Santos, M. W. Wassen, J. C. Camargo García, I. Llorca Fernandez, L. Verkuil, A.

Scheper, M. Steenhuis, and P. A. Verweij. 2021. "Ecosystem Services Trajectories in Coffee Agroforestry in Colombia over 40 Years." *Ecosystem Services* 48 (January): 101246. https://doi.org/10.1016/j.ecoser.2021.101246.

Okunda, T., M. Suzuki, N. Adachi, E. S. Quah, N. A. Hussein, and N. Manokaran. 2003. "Effect of Selective Logging on Canopy and Stand Structure and Tree Species Composition in a Lowland Dipterocarp Forest in Peninsular Malaysia." *Forest Ecology and Management* 175: 297–320. https://doi.org/https://doi.org/10.1016/S0378-1127(02)00137-8.

Orefice, Joseph, Richard G. Smith, John Carroll, Heidi Asbjornsen, and Daniel Kelting. 2017. "Soil and Understory Plant Dynamics during Conversion of Forest to Silvopasture, Open Pasture, and Woodlot." *Agroforestry Systems* 91 (4): 729–39. https://doi.org/10.1007/s10457-016-0040-y.

Pereira, Rodrigo, Johan Zweede, Gregory P. Asner, and Michael Keller. 2002. "Forest Canopy Damage and Recovery in Reduced-Impact and Conventional Selective Logging in Eastern Para, Brazil." *Forest Ecology and Management* 168 (1–3): 77–89. https://doi.org/10.1016/S0378-1127(01)00732-0.

Perez-Alvarez, Ricardo, Julián Chará, Lauren D. Snyder, Michelle Bonatti, Stefan Sieber, and Emily A. Martin. 2023. "Global Meta-Analysis Reveals Overall Benefits of Silvopastoral Systems for Biodiversity." *BioRxiv* 2023 (12): 2023.07.30.551160. iorxiv.org/content/10.1101/2023.07.30.551160v1%0Ahttps://www.biorxiv.org/content/10.1101/2023.07.30.551160v1.abstract.

Perry, Jitka, Bohdan Lojka, Lourdes G.Quinones Ruiz, Patrick Van Damme, Jakub Houška, and Eloy Fernandez Cusimamani. 2016. "How Natural Forest Conversion Affects Insect Biodiversity in the Peruvian Amazon: Can Agroforestry Help?" *Forests* 7 (4): 1–13. https://doi.org/10.3390/f7040082.

Perry, Kayla I., Kimberly F. Wallin, John W. Wenzel, and Daniel A. Herms. 2018. "Forest Disturbance and Arthropods: Small-Scale Canopy Gaps Drive Invertebrate Community Structure and Composition." *Ecosphere* 9 (10): 1–19. https://doi.org/10.1002/ecs2.2463.

Przepióra, Fabian, Jan Loch, and Michał Ciach. 2020. "Bark Beetle Infestation Spots as Biodiversity Hotspots: Canopy Gaps Resulting from Insect Outbreaks Enhance the Species Richness, Diversity and Abundance of Birds Breeding in Coniferous Forests." *Forest Ecology and Management* 473: 118280. https://doi.org/10.1016/j.foreco.2020.118280.

Savilaakso, Sini, Claude Garcia, John Garcia-Ulloa, Jaboury Ghazoul, Martha Groom, Manuel R. Guariguata, Yves Laumonier, et al. 2014. "Systematic Review of Effects on Biodiversity from Oil Palm Production." *Environmental Evidence* 3 (1). https://doi.org/10.1186/2047-2382-3-4.

Schwartz, Gustavo, Marielos Peña-Claros, José C.A. Lopes, Godefridus M.J. Mohren, and Milton Kanashiro. 2012. "Mid-Term Effects of Reduced-Impact Logging on the Regeneration of Seven Tree Commercial Species in the Eastern Amazon." *Forest Ecology and Management* 274: 116–25. https://doi.org/10.1016/j.foreco.2012.02.028.

Tudge, Sophie Jane, Zoe M. Harris, Richard J. Murphy, Andy Purvis, and Adriana De Palma. 2023. "Global Trends in Biodiversity with Tree Plantation Age." *Global Ecology and Conservation* 48 (August): e02751. https://doi.org/10.1016/j.gecco.2023.e02751.

Wang, Chao, Weiwei Zhang, Xiaona Li, and Juying Wu. 2022. "A Global Meta-Analysis of the Impacts of Tree Plantations on Biodiversity." *Global Ecology and Biogeography* 31 (3): 576–87. https://doi.org/10.1111/geb.13440.

Xu, H., Y. Li, S. Liu, R. Zang, F. He, and J. R. Spence. 2015. "Partial Recovery of a Tropical Rain Forest a Half-Century after Clear-Cut and Selective Logging." *Journal of Applied Ecology* 52 (4): 1044–52. https://doi.org/10.1111/1365-2664.12448.

Yahya, Muhammad Syafiq, Sharifah Nur Atikah, Izereen Mukri, Ruzana Sanusi, Ahmad Razi

Norhisham, and Badrul Azhar. 2022. “Agroforestry Orchards Support Greater Avian Biodiversity than Monoculture Oil Palm and Rubber Tree Plantations.” *Forest Ecology and Management* 513 (March): 120177. https://doi.org/10.1016/j.foreco.2022.120177.

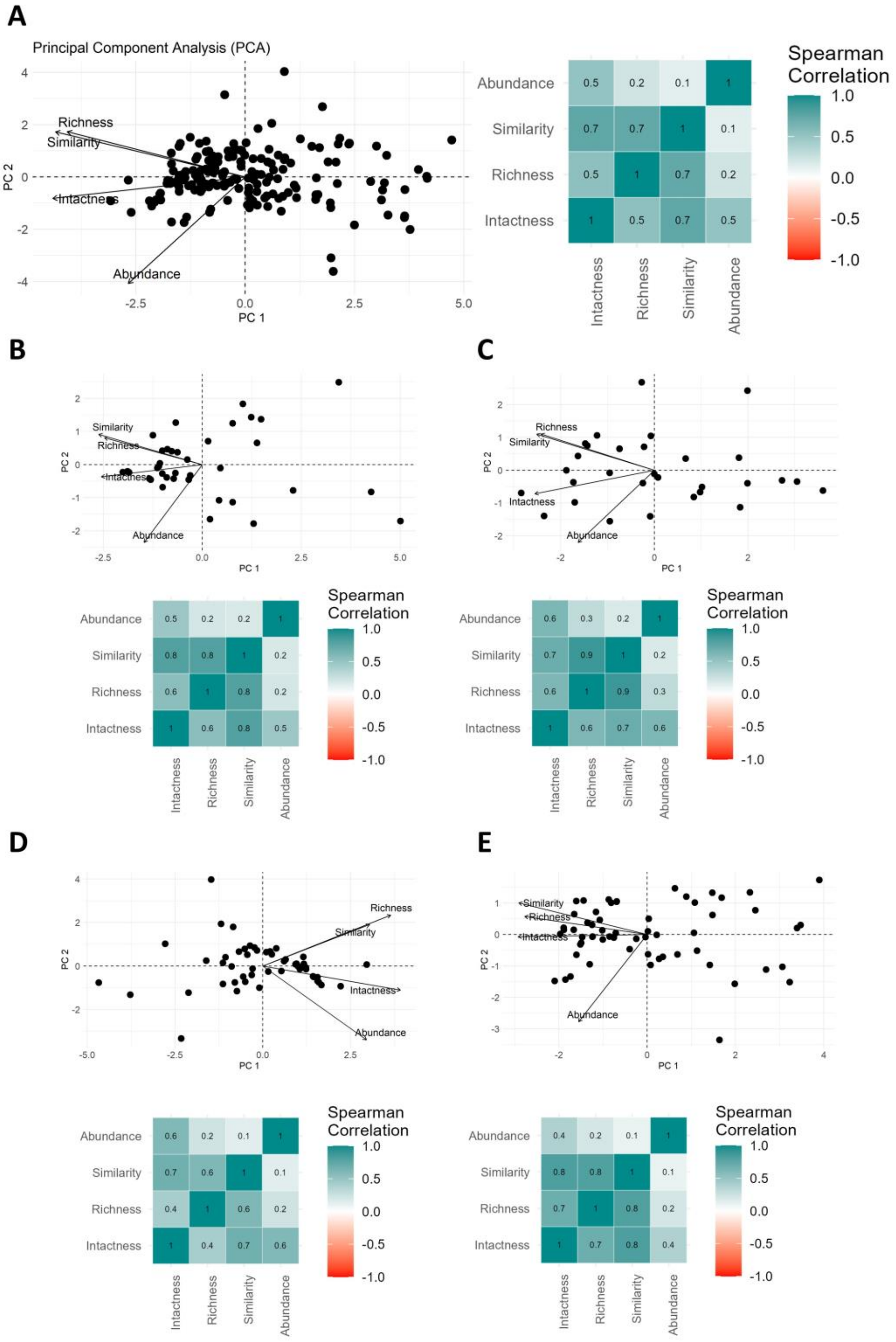

Figure S1. Principal component analyses and Spearman correlation plots for all response ratios of all management systems for animals altogether (A), birds (B), herpetofauna (C), mammals (D) and insects (E). The results indicate that intactness, relative richness, similarity and relative total abundance capture distinct, yet positively correlated aspects of animal biodiversity across taxonomic groups.

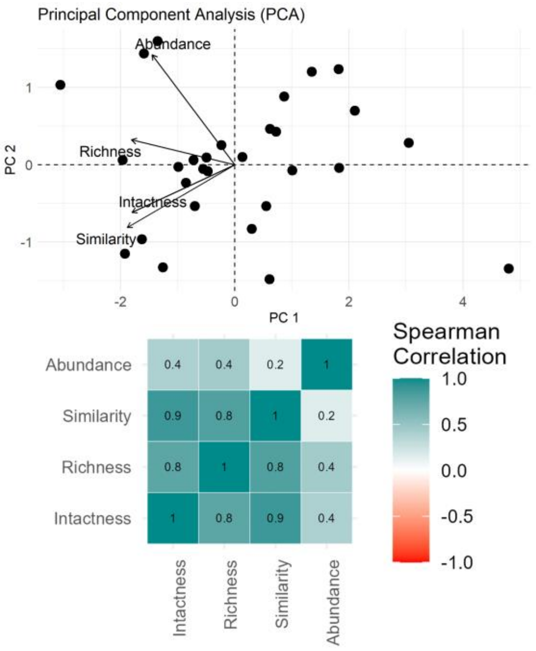

Figure S2. Principal component analyses and Spearman correlation plots for all response ratios of all management systems for plant biodiversity. The results indicate that intactness, relative richness, similarity and relative total abundance capture distinct, yet positively correlated aspects of plant biodiversity.

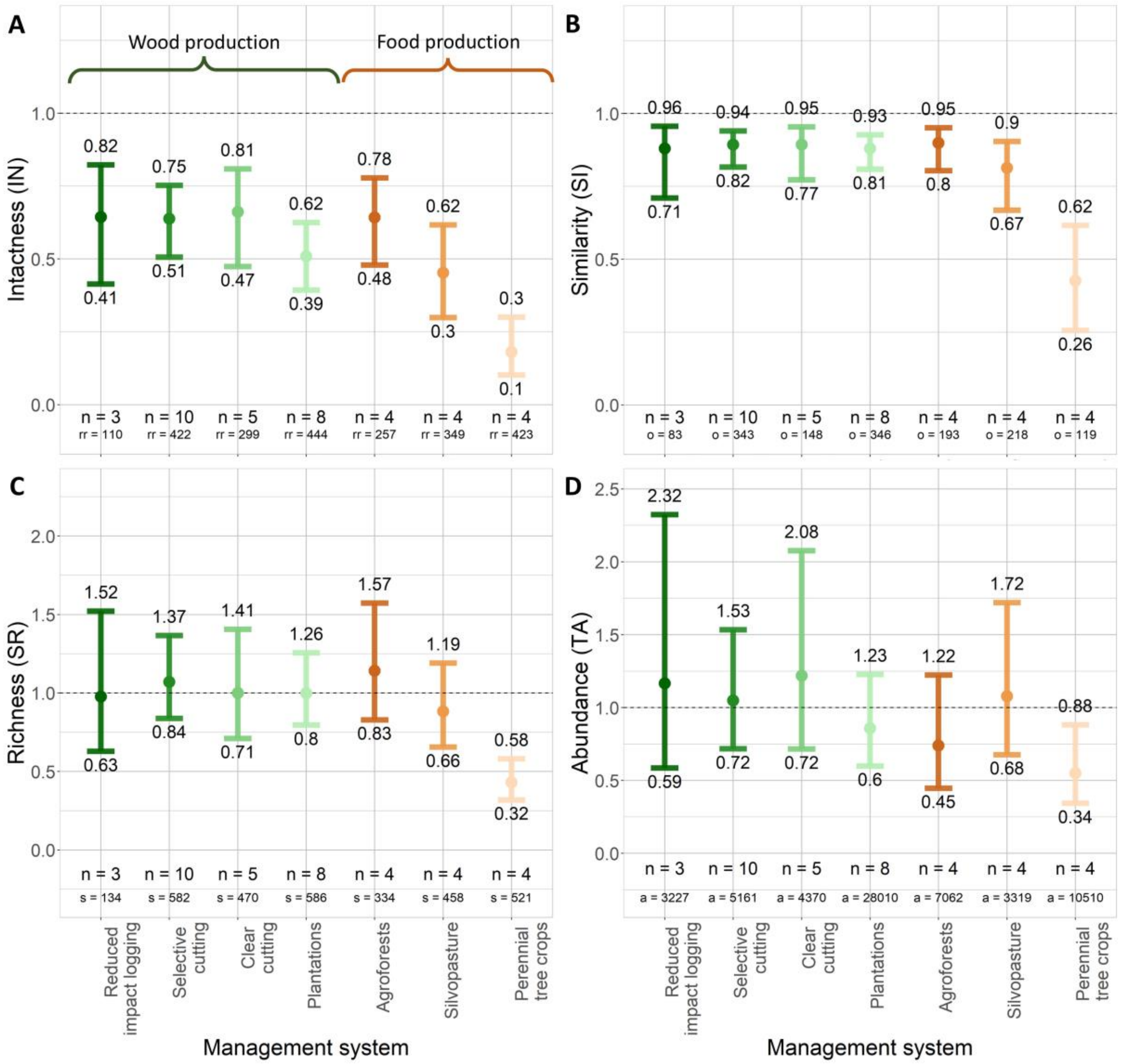

Figure S3. Effects of forest management on bird biodiversity, expressed in four indicators: intactness (IN) (A), similarity (SI) (B), relative richness (SR) (C), and relative total abundance (TA) (D). Each colored dot shows the back-transformed estimate we derived from our mixed models, and the bars indicate the 95% confidence interval of the estimate. The dotted line indicates the reference level biodiversity. n = number of studies included per management type, rr = number of response values used to compute intactness, o = number of overlapping species between managed and reference forest sites, s = number of species occurring across the managed and reference sites, a = number of abundance values used to compute relative total abundance.

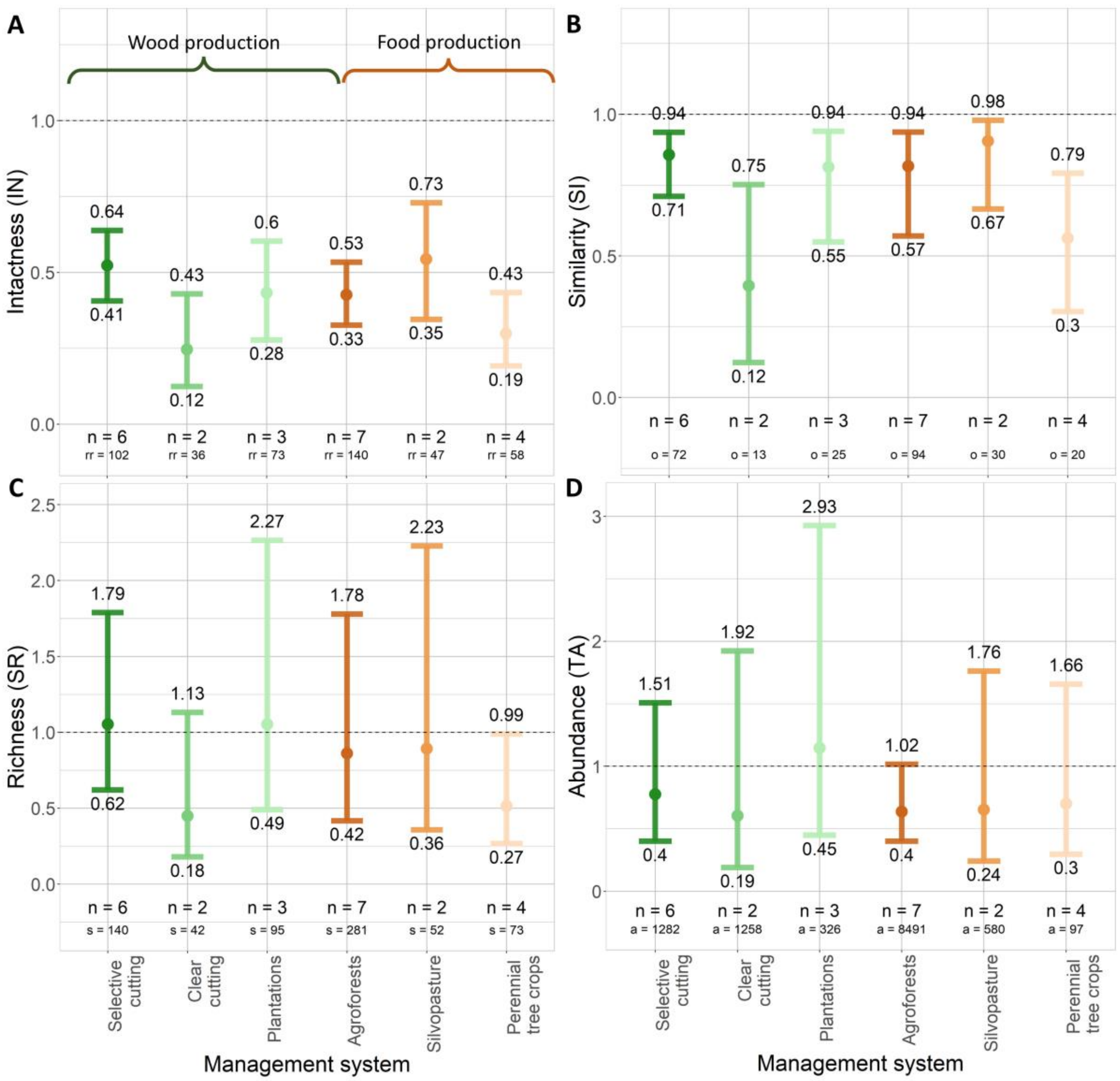

Figure S4. Effects of forest management on herpetofauna biodiversity, expressed in four indicators: intactness (IN) (A), similarity (SI) (B), relative richness (SR) (C), and relative total abundance (TA) (D). Each colored dot shows the back-transformed estimate we derived from our mixed models, and the bars indicate the 95% confidence interval of the estimate. The dotted line indicates the reference level biodiversity. n = number of studies included per management type, rr = number of response values used to compute intactness, o = number of overlapping species between managed and reference forest sites, s = number of species occurring across the managed and reference sites, a = number of abundance values used to compute relative total abundance.

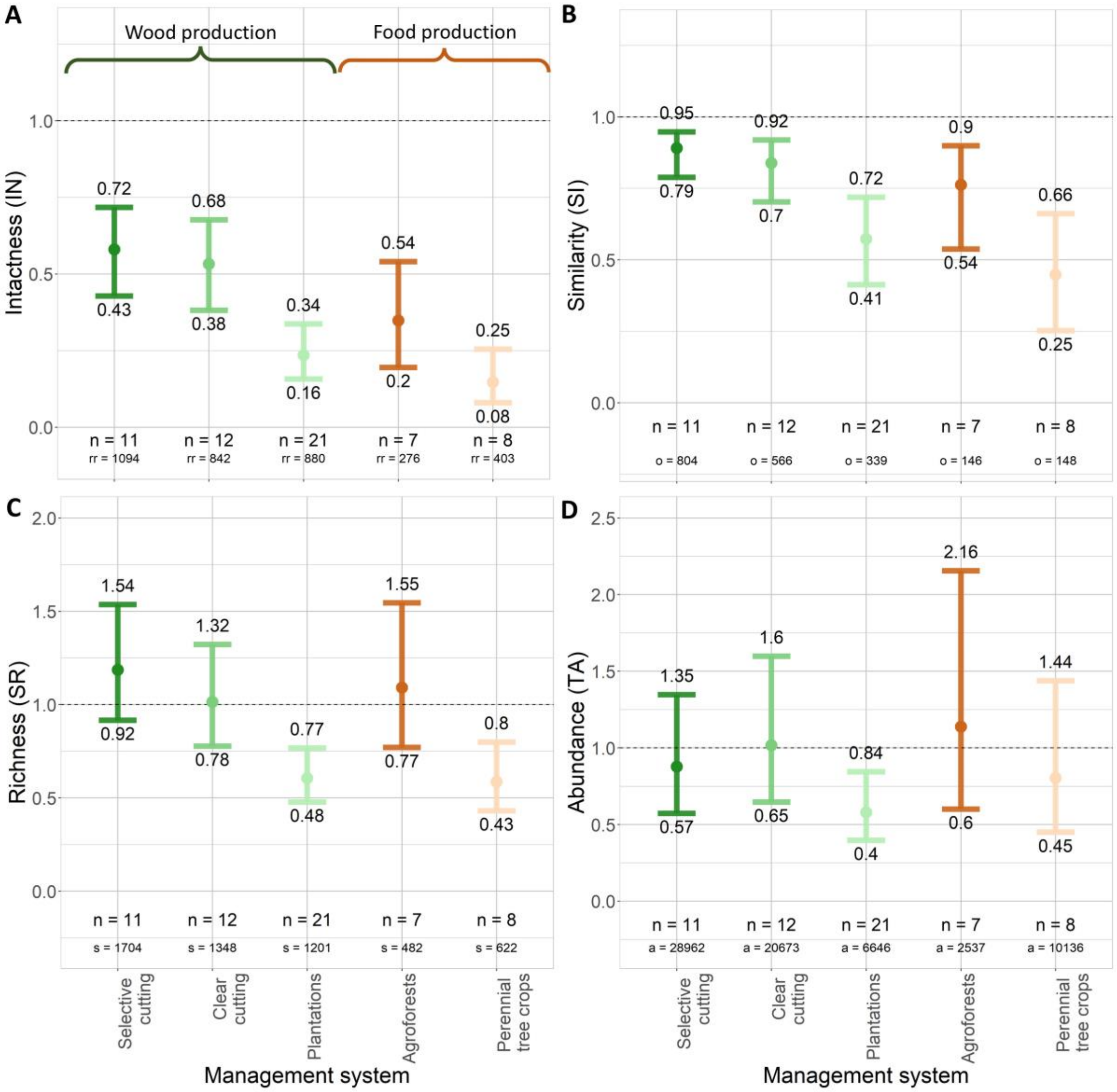

Figure S5. Effects of forest management on insect biodiversity, expressed in four indicators: intactness (IN) (A), similarity (SI) (B), relative richness (SR) (C), and relative total abundance (TA) (D). Each colored dot shows the back-transformed estimate we derived from our mixed models, and the bars indicate the 95% confidence interval of the estimate. The dotted line indicates the reference level biodiversity.n = number of studies included per management type, rr = number of response values used to compute intactness, o = number of overlapping species between managed and reference forest sites, s = number of species occurring across the managed and reference sites, a = number of abundance values used to compute relative total abundance. The dotted line indicates the reference level biodiversity.

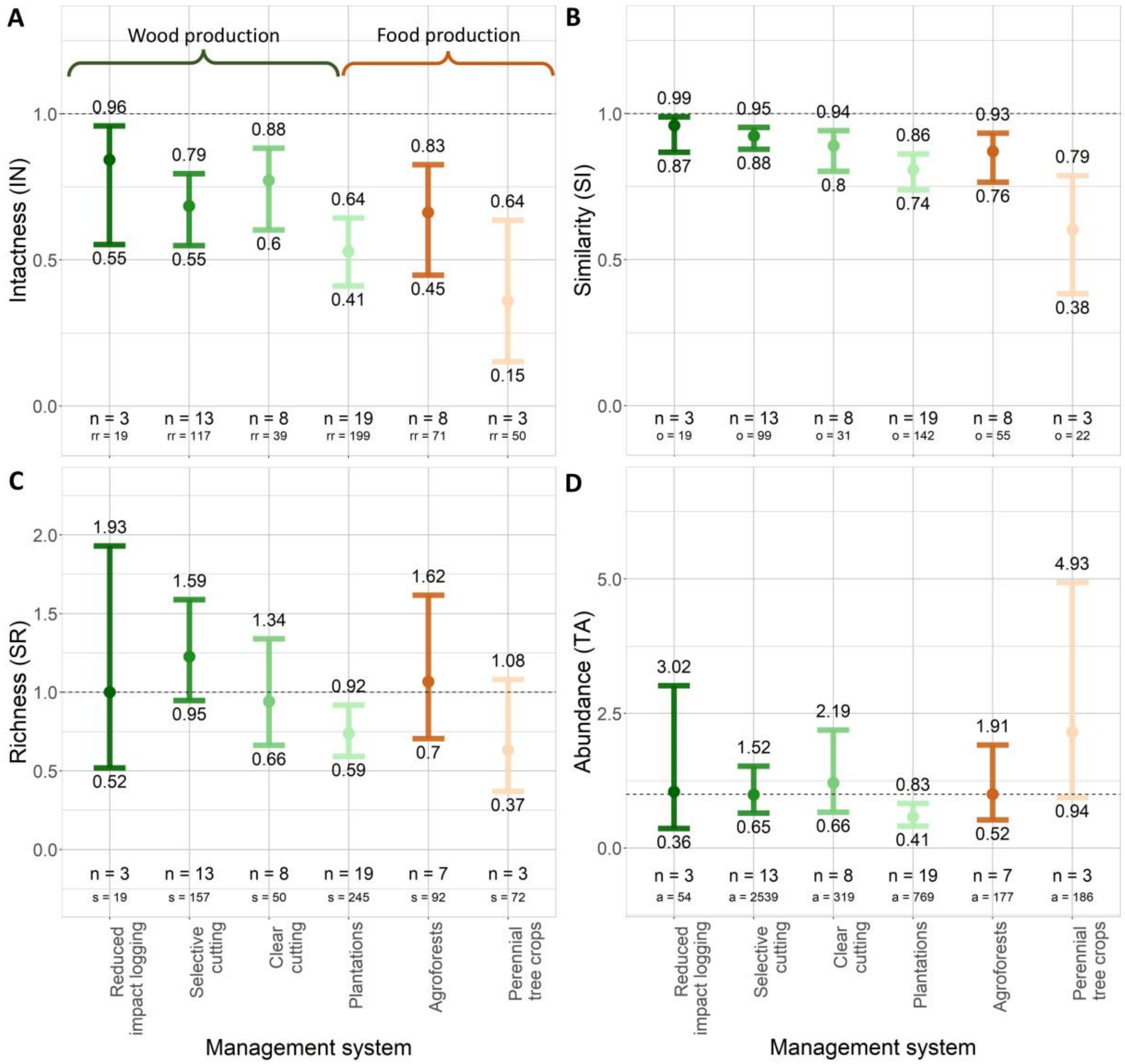

Figure S6. Effects of forest management on mammal biodiversity, expressed in four indicators: intactness (IN) (A), similarity (SI) (B), relative richness (SR) (C), and relative total abundance (TA) (D). Each colored dot shows the back-transformed estimate we derived from our mixed models, and the bars indicate the 95% confidence interval of the estimate. The dotted line indicates the reference level biodiversity. n = number of studies included per management type, rr = number of response values used to compute intactness, o = number of overlapping species between managed and reference forest sites, s = number of species occurring across the managed and reference sites, a = number of abundance values used to compute relative total abundance.
